# Photoredox catalysis in nickel-catalyzed C–H functionalization

**DOI:** 10.3762/bjoc.17.143

**Published:** 2021-08-31

**Authors:** Lusina Mantry, Rajaram Maayuri, Vikash Kumar, Parthasarathy Gandeepan

**Affiliations:** 1 Department of Chemistry, Indian Institute of Technology Tirupati, Tirupati – Renigunta Road, Settipalli Post, Tirupati, Andhra Pradesh 517506, India

**Keywords:** C–H activation, functionalization, nickel, photocatalysts, photoredox, visible light

## Abstract

Catalytic C‒H functionalization has become a powerful strategy in organic synthesis due to the improved atom-, step- and resource economy in comparison with cross-coupling or classical organic functional group transformations. Despite the significant advances in the metal-catalyzed C‒H activations, recent developments in the field of metallaphotoredox catalysis enabled C‒H functionalizations with unique reaction pathways under mild reaction conditions. Given the relative earth-abundance and cost-effective nature, nickel catalysts for photoredox C‒H functionalization have received significant attention. In this review, we highlight the developments in the field of photoredox nickel-catalyzed C‒H functionalization reactions with a range of applications until summer 2021.

## Introduction

During the last decades, transition-metal-catalyzed transformations have become one of the most reliable and basic tools for designing and manufacturing biologically relevant molecules and functional materials [1–4]. The formation of highly chemo-, regio-, and stereoselective products with excellent yields is the key reason for transition-metal catalysis as a reliable strategy in modern organic synthesis. Palladium-catalyzed cross-coupling reactions such as Mizoroki–Heck [5–8], Suzuki–Miyaura [9–11], Buchwald–Hartwig [12–13], Negishi [14–15], Migita–Stille [16], Sonogashira [17], among others [18–20], significantly changed the design of synthetic routes for modern pharmaceuticals [21–22]. Over the past two decades, nickel has emerged as an attractive alternative to palladium due to its relative earth-abundance, less toxicity, and inexpensiveness.

**Scheme 1:**
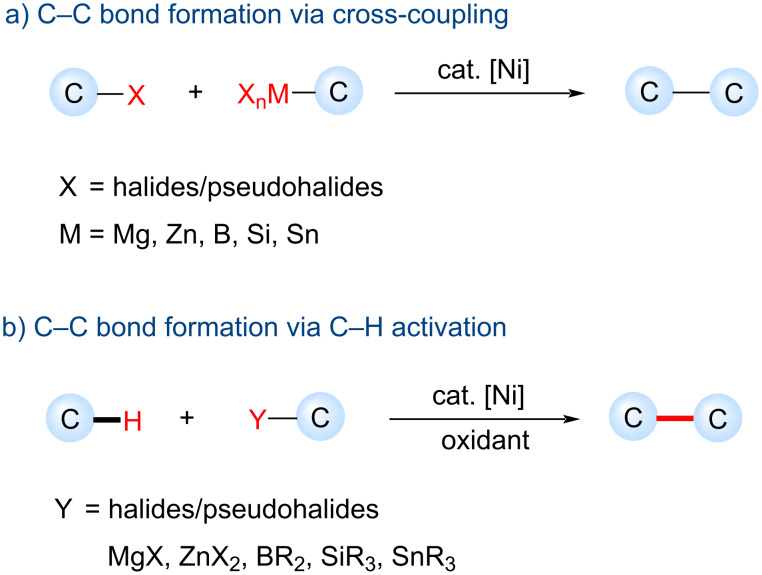
Nickel-catalyzed cross-coupling versus C‒H activation.

Despite the fact that the nickel-catalyzed cross-coupling reactions represent a powerful tool in organic synthesis, they generally require prefunctionalized starting materials, which significantly affect the reaction's atom economy and produce inorganic, organometallic salt wastes [23–25]. During the last decade, the oxidative functionalization of inert C‒H into carbon–carbon (C‒C) and carbon–heteroatom bonds for the construction of complex organic molecules by nickel catalysis significantly improved the atom-, step-, and resource economy by avoiding the substrate prefunctionalizations (Scheme 1) [26–30]. The nickel-catalyzed oxidative C‒H functionalization often requires relatively high catalyst loadings, directing groups, high reaction temperatures (100–160 °C), stoichiometric additives, or oxidants such as peroxide or silver salts that can be undesirable for large scale synthesis.

**Figure 1:**
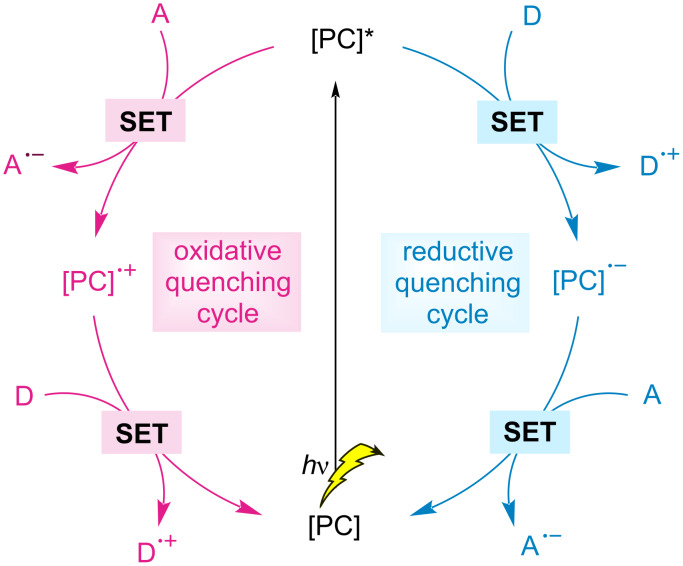
Oxidative and reductive quenching cycles of a photocatalyst. [PC] = photocatalyst, A = acceptor, D = donor.

Recently, photoredox dual catalysis has witnessed significant developments, which enables a diverse range of previously inaccessible organic transformations in milder reaction conditions [31–40]. Here, by absorbing visible light, a photocatalyst can function as a single-electron redox mediator through an oxidative or reductive quenching cycle (Figure 1), thereby facilitating redox-neutral transformations in the absence of stoichiometric oxidants/reductants. Given the tendency of nickel to mediate the reactions via Ni(0), Ni(I), Ni(II), and Ni(III) intermediates by both giving and accepting a single electron from a photocatalyst or combined with radical species [41–43], a wide variety of reactions have been discovered. Within a remarkable renaissance of photoredox dual catalysis, nickel/photoredox catalysis has recently been identified as a viable C‒H functionalization tool under milder reaction conditions [40,44–47]. In this review, we highlight the developments in C–H activation enabled by nickel photocatalysis.

## Review

### Arylation

**Scheme 2:**
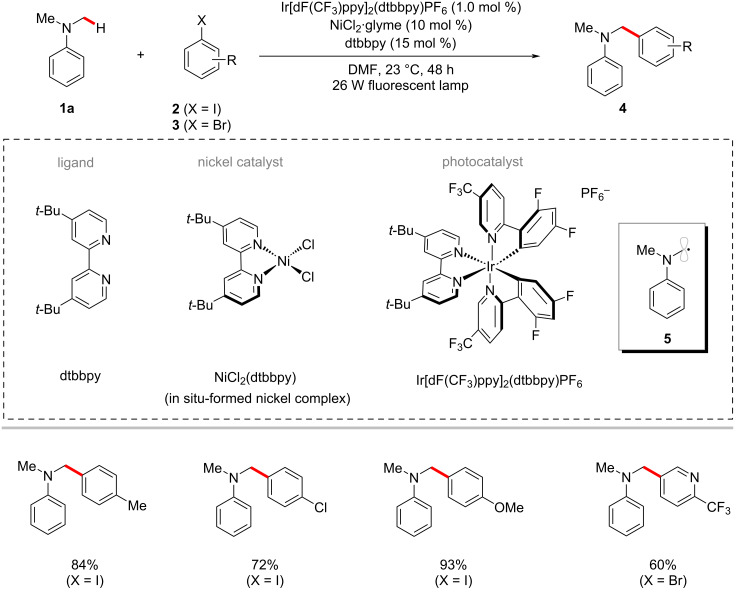
Photoredox nickel-catalyzed C(sp^3^)–H arylation of dimethylaniline (**1a**).

The arylation of C(sp^3^)‒H bonds constitutes a potential tool for the rapid diversification of simple organic molecules into valuable scaffolds [48–52]. In 2014, Doyle, MacMillan and co-workers demonstrated an inspiring C(sp^3^)‒H arylation of dimethylaniline (**1a**) with a variety of aryl halides using the photoredox nickel catalysis [53]. Here, the combination of the iridium photocatalyst Ir[dF(CF_3_)ppy]_2_(dtbbpy)PF_6_ and the commercially available nickel catalyst NiCl_2_·glyme were found to be suitable to achieve the transformation in satisfactory yields under visible light irradiation (Scheme 2). The authors hypothesized that the key α-nitrogen carbon-centered radical **5** could be generated via a photoredox-driven *N*-phenyl oxidation and α-C–H deprotonation sequence from dimethylaniline (**1a**) and should intercept with the nickel catalytic cycle to result in the desired products **4**.

**Scheme 3:**
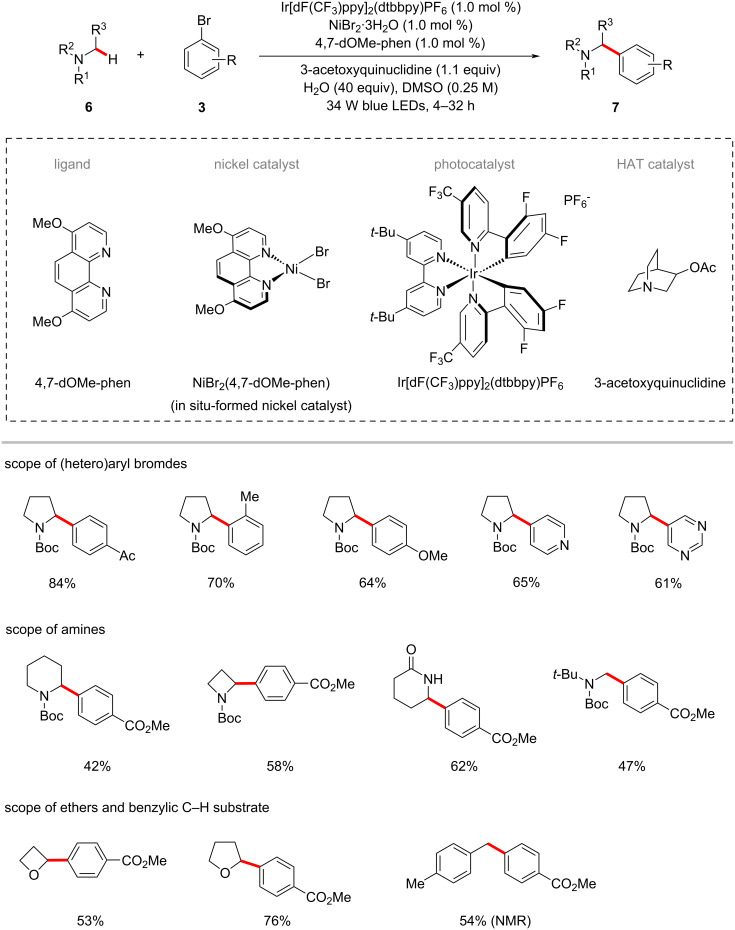
Photoredox nickel-catalyzed arylation of α-amino, α-oxy and benzylic C(sp^3^)‒H bonds with aryl bromides.

In another work by the same laboratory, a strategy for the arylation of α-amino C(sp^3^)–H bonds in various acyclic and cyclic amine compounds **6** was realized using photoredox-mediated hydrogen atom transfer (HAT) and nickel catalysis [54]. The catalytic system consisting of iridium photocatalyst Ir[dF(CF_3_)ppy]_2_(dtbbpy)PF_6_, nickel catalyst NiBr_2_·3H_2_O, ligand 4,7-dimethoxy-1,10-phenanthroline (4,7-dOMe-phen), and 3-acetoxyquinuclidine was found to be optimal to afford the desired α-amino C–C coupled products **7** (Scheme 3). It is worth noting that 3-acetoxyquinuclidine serves as both the HAT catalyst and the base in this reaction system. Furthermore, several cyclic and acyclic amine **6** substrates were used as C‒H nucleophile coupling partners for (hetero)aryl bromides **3**. Two additional examples for the photoredox nickel-catalyzed arylation of α-oxy C–H bonds of tetrahydrofuran (THF) and oxetane were also shown. Further, the catalytic system also proved compatible for the C‒H arylation of the benzylic system.

**Figure 2:**
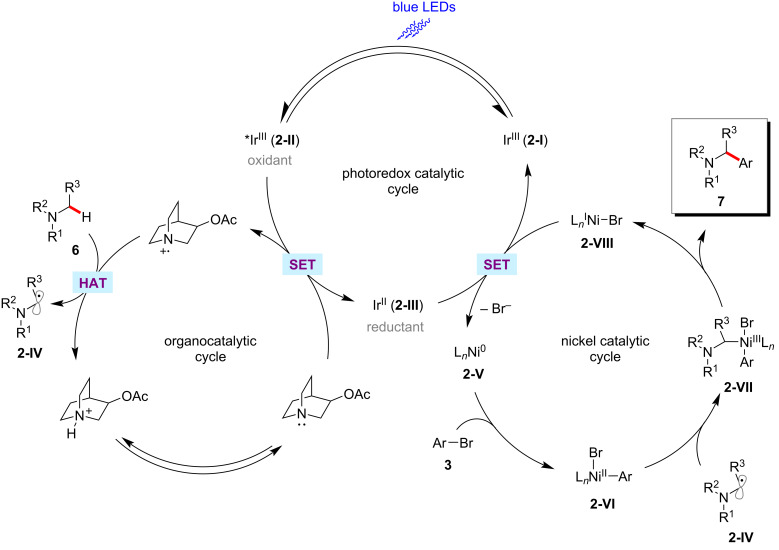
Proposed catalytic cycle for the photoredox-mediated HAT and nickel catalysis enabled C(sp^3^)‒H arylation.

As shown in Figure 2 [54], the mechanism for the transformation is proposed to involve the generation of nucleophilic α-amino radicals **2-IV** via a photoredox-mediated HAT process. At the same time, the in situ generated nickel(0) species **2-V** by a SET process would undergo oxidative addition into aryl bromide **3**, resulting in the electrophilic nickel(II)–aryl intermediate **2-VI**. The rapid coupling of nickel(II) species **2-VI** and radical species **2-IV** forms a nickel(III) intermediate **2-VII**, which undergoes a reductive elimination to afford the desired product **7** and the nickel(I) species **2-VIII**. The SET reduction of **2-VIII** by the iridium(II) species **2-III** regenerates the nickel(0) catalyst **2-V** and the iridium(III) photocatalyst **2-I**.

**Scheme 4:**
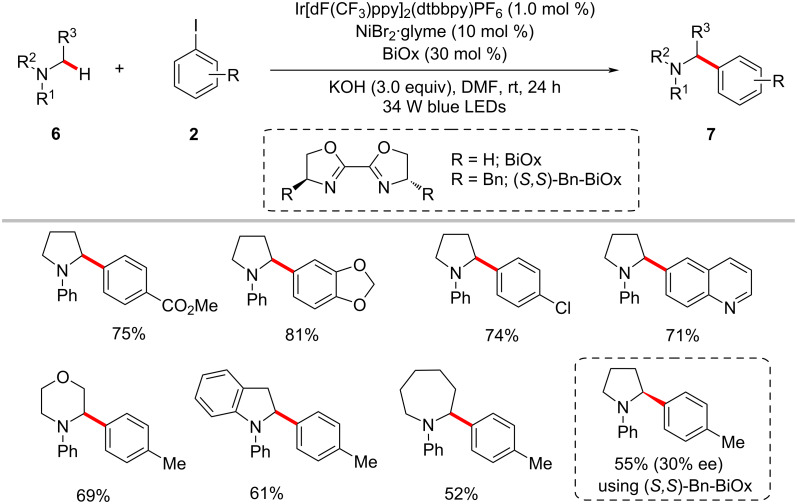
Photoredox arylation of α-amino C(sp^3^)‒H bonds with aryl iodides.

Subsequently, Ahneman and Doyle reported a related process for the synthesis of a variety of benzylic amines **7** by the arylation of α-amino C(sp^3^)‒H bonds with aryl iodides **2** involving photoredox nickel catalysis (Scheme 4) [55]. In this protocol, bis(oxazoline) (BiOx) was identified as the suitable ligand instead of the commonly used bipyridyl ligand (vide supra). Notably, the use of the chiral (*S,S*)-Bn-BiOx ligand resulted in a moderate enantioinduction in the C‒H arylation product.

**Figure 3:**
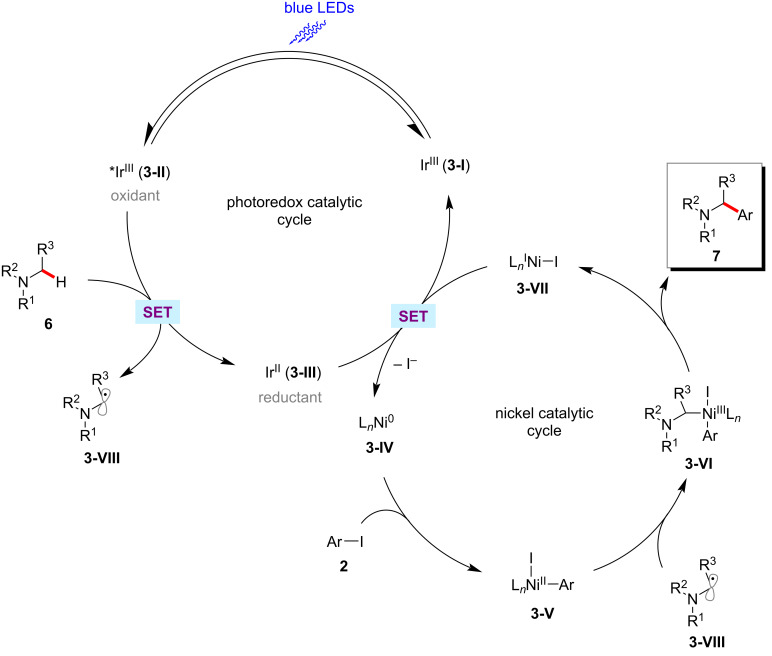
Proposed mechanism for photoredox nickel-catalyzed α-amino C‒H arylation with aryl iodides.

The authors proposed a catalytic cycle to account for the photoredox nickel-catalyzed C(sp^3^)‒H arylation as shown in Figure 3 [55]. Thus, the in situ-generated nickel(0) **3-IV** undergoes an oxidative addition with the aryl iodide **2** to form the nickel(II)–aryl complex **3-V**. The photoredox-generated nucleophilic α-amino radical **3-VIII** readily combines with the nickel(II) species **3-V** to generate nickel(III) intermediate **3-VI**, which results in the cross-coupled product **7** upon reductive elimination. The SET event between the reduced photocatalyst **3-III** and the nickel(0) species **3-IV** regenerates both catalysts simultaneously.

**Scheme 5:**
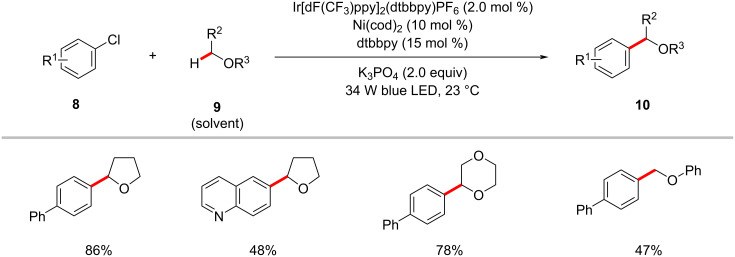
Nickel-catalyzed α-oxy C(sp^3^)−H arylation of cyclic and acyclic ethers.

**Figure 4:**
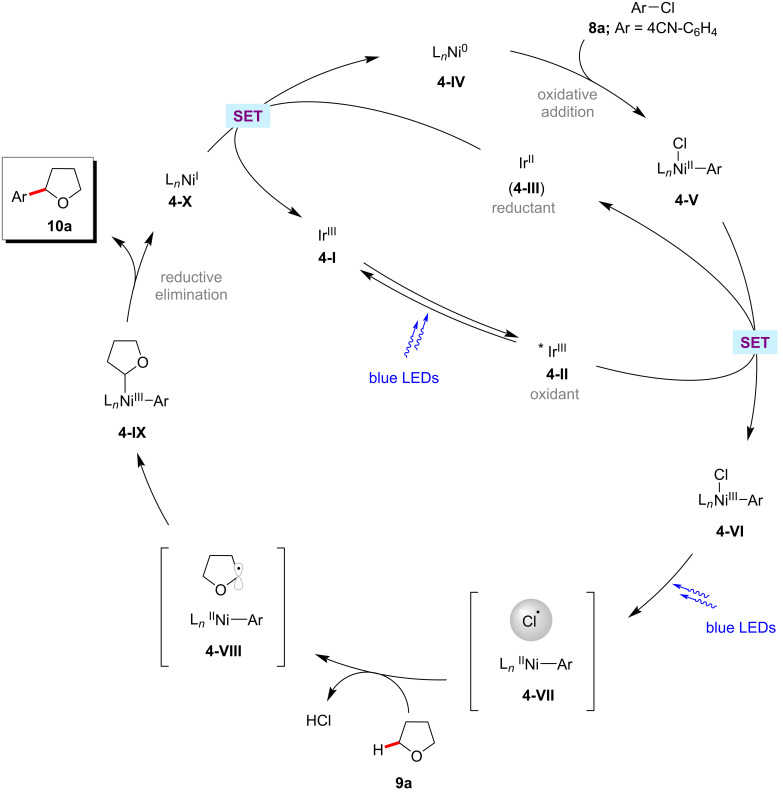
Proposed catalytic cycle for the C(sp^3^)−H arylation of cyclic and acyclic ethers.

Further studies by the Doyle group established the α-oxy C(sp^3^)−H arylation of cyclic and acyclic ethers **9** with aryl chlorides **8** under photoredox nickel catalysis (Scheme 5) [56]. Here, aryl chlorides **8** serve as cross-coupling partners and the chlorine radical source, which rapidly abstracts an α-oxy C(sp^3^)−H of the ethers to form the key α-oxyalkyl radical intermediate. Notably, the photocatalytic conditions proved suitable for the benzylic C(sp^3^)−H and unactivated alkane cyclohexane C‒H arylations. The catalytic cycle is proposed to involve the oxidative addition of nickel(0) **4-IV** into an aryl chloride **8a** to form nickel(II) intermediate **4-V** (Figure 4) [56]. The SET oxidation of **4-V** by the photoexcited iridium(III) photocatalyst **4-II** results in the nickel(III) species **4-VI**. Photolysis of **4-VI** generates a chloride radical, which rapidly abstracts the α-oxy C(sp^3^)−H of the ether to provide the alkyl radical species. The alkyl radical rebound to **4-VIII** produces the nickel(III) species **4-IX**, which undergoes reductive elimination to release the desired product **10a**.

**Scheme 6:**
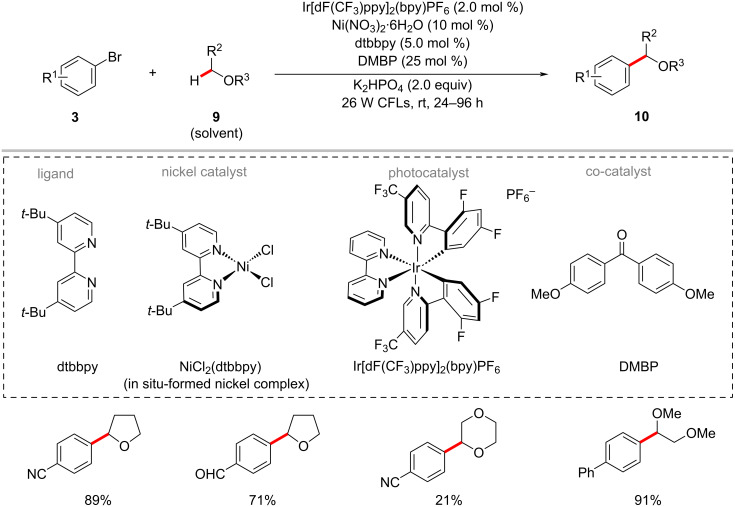
Photochemical nickel-catalyzed C–H arylation of ethers.

**Figure 5:**
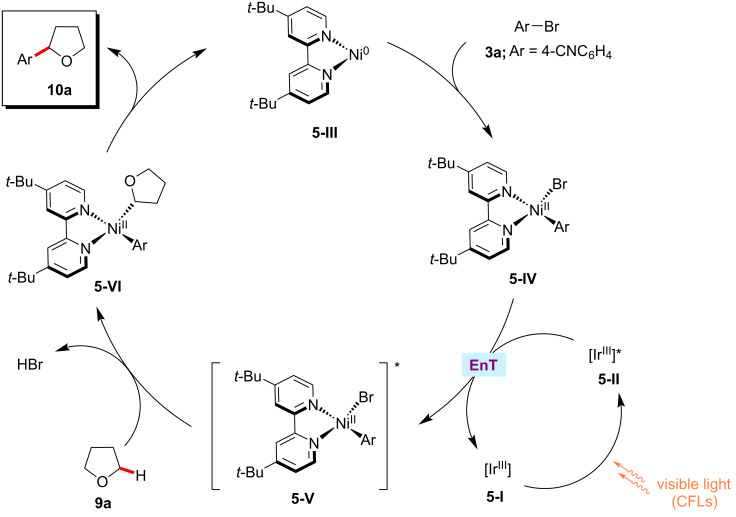
Proposed catalytic cycle for the nickel-catalyzed arylation of ethers with aryl bromides.

Concurrently, Molander and co-workers also reported a related nickel-catalyzed arylation of α-heteroatom-substituted or benzylic C(sp^3^)‒H bonds by aryl bromides **3** at room temperature using an iridium photocatalyst, substoichiometric 4,4′-dimethoxybenzophenone (DMBP) additives, and visible light (Scheme 6) [57]. A variety of cyclic and acyclic ethers **9** reacted with (hetero)aryl bromides **3** under the mild reaction conditions to give the desired products **10** in moderate to good yields, however, with longer reaction times (24–96 h). The authors proposed a catalytic cycle to account for the mode of operation as depicted in Figure 5 [57]. Thus, the in situ-generated nickel(0) complex **5-III** undergoes oxidative addition into aryl bromide **3a** to form nickel(II) complex **5-IV**. The triplet–triplet energy transfer from the excited photocatalyst to the **5-IV** complex resulted in excited **5-V**. Subsequently, the homolysis of the Ni–Br bond in **5-V** followed by a HAT process results in species **5-VI**. The nickel–alkyl–aryl complex **5-VI** undergoes reductive elimination to release the desired product **10a** and regenerates the active nickel(0) catalyst **5-III**.

**Scheme 7:**
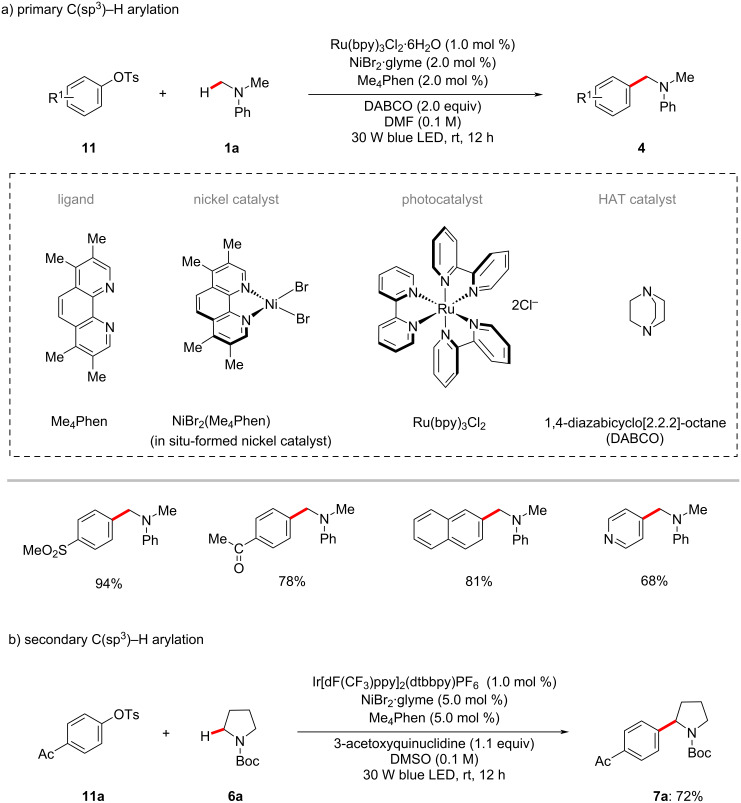
Nickel-catalyzed α-amino C(sp^3^)‒H arylation with aryl tosylates.

The synthetic utility of the photoredox nickel-catalyzed C‒H arylation was further elaborated to include C‒O electrophiles which could be readily derived from phenols, as disclosed by the Yu group [58]. Hence, they reported an arylation protocol for α-amino- and α-oxy C(sp^3^)‒H bonds with aryl tosylates/triflates **11**. The relatively less expensive ruthenium photocatalyst Ru(bpy)_3_Cl_2_·6H_2_O was found to be optimal for primary C(sp^3^)‒H arylations (Scheme 7a), whereas Ir[dF(CF_3_)ppy]_2_(dtbbpy)PF_6_ was the effective photocatalyst for the arylation of secondary C(sp^3^)‒H bonds (Scheme 7b).

**Scheme 8:**
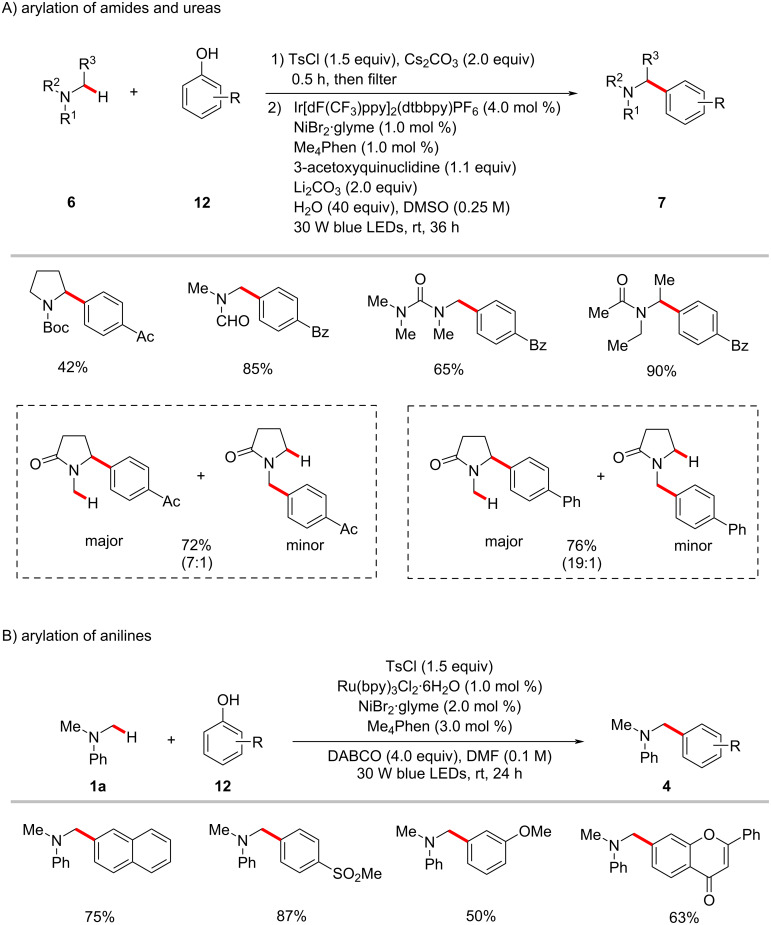
Arylation of α-amino C(sp^3^)‒H bonds by in situ generated aryl tosylates from phenols.

In a subsequent report, Yu and co-workers also realized the arylation of α-amino C(sp^3^)‒H bonds with aryl tosylates **11** generated in situ from phenols **12** and *p*-toluenesulfonyl chloride (TsCl) [59–60]. The combination of visible-light-photoredox catalysis, hydrogen-atom-transfer catalysis, and nickel catalysis enables these protocols at room temperature with ample substrate scope (Scheme 8). Unsymmetrical amine substrates favored arylation at the methylene C‒H over methyl C‒H with good regioselectivity [59].

**Scheme 9:**
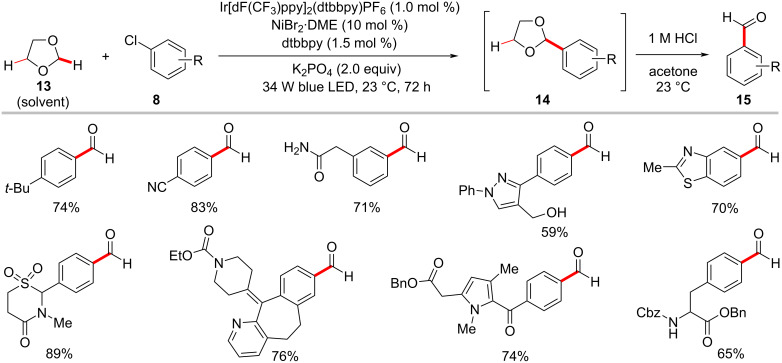
Formylation of aryl chlorides through redox-neutral 2-functionalization of 1,3-dioxolane (**13**).

In 2017, Doyle utilized the photoredox nickel catalysis approach for the formylation of aryl chlorides **8** through selective 2-functionalization of 1,3-dioxolane (**13**) followed by a mild acidic workup (Scheme 9) [61]. Here, the photocatalyst Ir[dF(CF_3_)ppy]_2_(dtbbpy)PF_6_ and nickel catalyst NiCl_2_·DME with dtbbpy as ligand, along with K_3_PO_4_ as base under irradiation with blue LEDs enabled the regioselective 2-functionalization of 1,3-dioxolane (**13**) with aryl chlorides **8**. It was found that the electron-deficient aryl chlorides resulted in better yields within shorter reaction times over the electron-rich substrates. A possible catalytic cycle was shown to account for the reaction mode, which is similar to that of Figure 4.

**Scheme 10:**
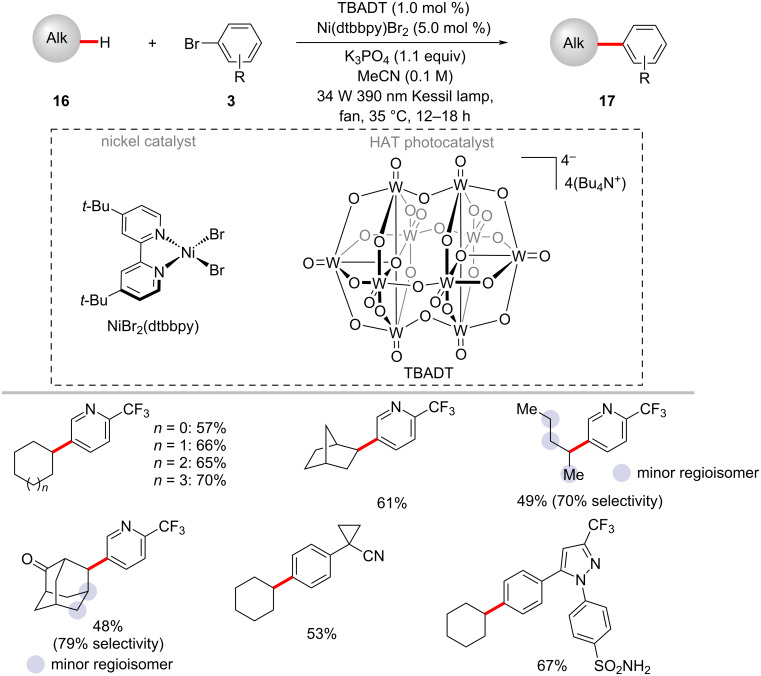
Photochemical C(sp^3^)–H arylation via a dual polyoxometalate HAT and nickel catalytic manifold.

**Figure 6:**
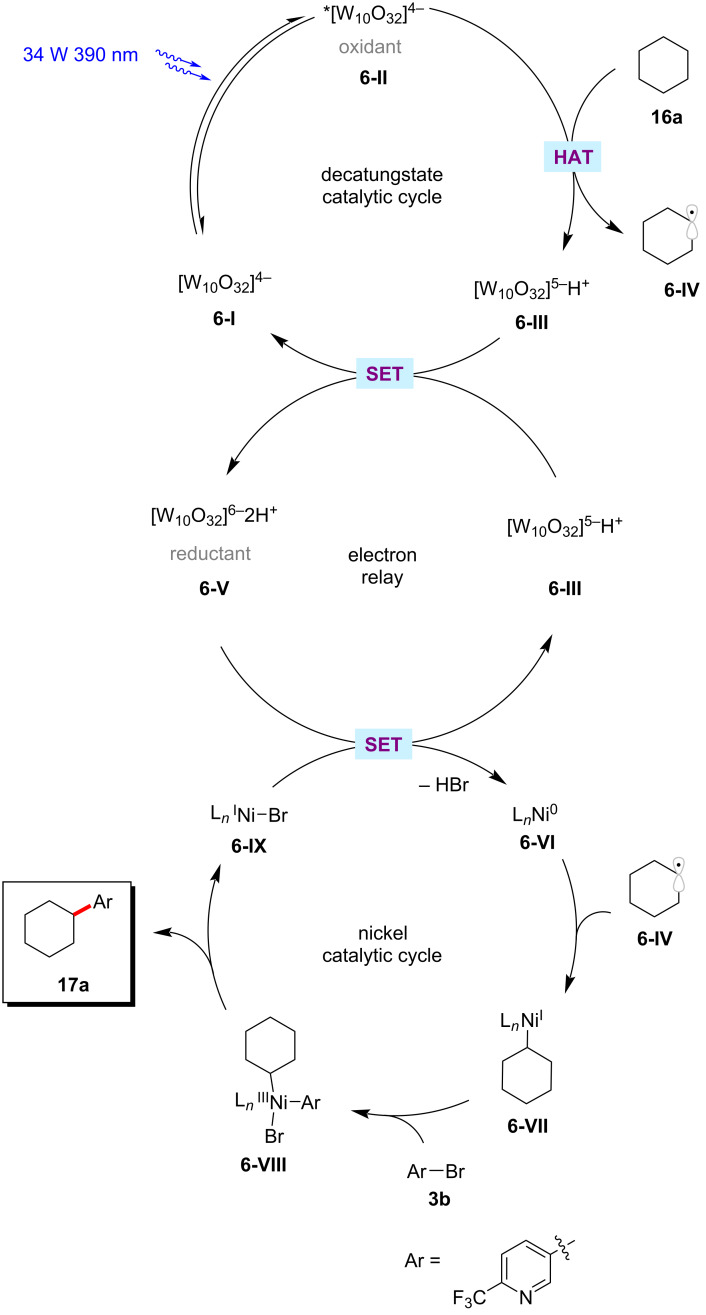
Proposed mechanism for C(sp^3^)–H arylation through dual polyoxometalate HAT and nickel catalytic manifold.

The robustness of the photoredox nickel catalysis was further demonstrated by a protocol for the direct arylation of inert aliphatic C‒H bonds [62]. Thus, MacMillan and co-workers employed tetrabutylammonium decatungstate [(W_10_O_32_)^4−^·4(Bu_4_N^+^)] (TBADT) as an efficient HAT photocatalyst to perform the desired C–H abstraction (Scheme 10) [62]. The catalytic reaction required near-ultraviolet light irradiation (Kessil 34 W 390 nm LEDs) and the exclusion of both oxygen and water to the success of the reaction. A variety of cyclic, acyclic, and bicyclic aliphatic systems **16** were arylated in moderate to good yields. This photochemical C–H arylation protocol was also suitable for functionalizing diverse primary and secondary benzylic C–H bonds. The authors proposed a mechanism for this chemo- and regioselective C‒H arylation as shown in Figure 6 [62]. The photoexcited decatungstate **6-II** undergoes a HAT process with alkyl substrate **16a** to form singly reduced decatungstate **6-III** and the carbon-centered radical **6-IV**. The active HAT photocatalyst **6-I** is regenerated by disproportionation of the singly reduced decatungstate **6-III**. At the same time, a nickel(0) species **6-VI** generated from the nickel(II) pre-catalyst by a SET process, captures the alkyl radical **6-IV** to furnish the nickel(I)–alkyl species **6-VII**. Subsequently, the nickel(I)–alkyl species **6-VII** undergoes oxidative addition into aryl bromide **3b** followed by a reductive elimination to provide the desired cross-coupled product **17a** and nickel(I) bromide complex **6-IX**. The final SET between this nickel(I) bromide species **6-IX** and the doubly reduced polyoxometalate **6-V** regenerates the active nickel(0) catalyst **6-VI** and reduced TBADT **6-I**. The authors also considered an alternative mechanism involving the oxidative addition of the nickel(0) catalyst **6-VI** to aryl bromide **3b**.

**Scheme 11:**
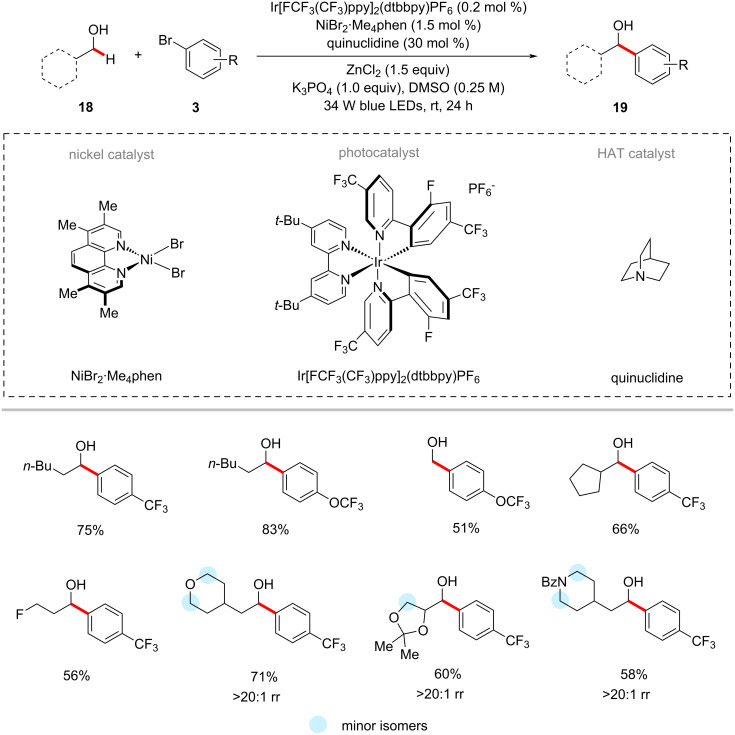
Photochemical nickel-catalyzed α-hydroxy C‒H arylation.

**Scheme 12:**
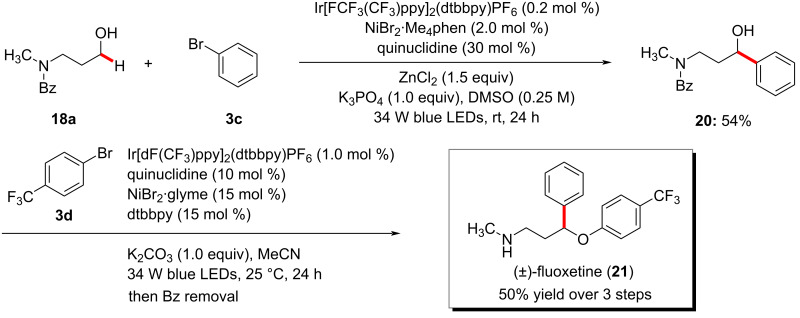
Photochemical synthesis of fluoxetine (**21**).

The photochemical nickel catalysis is not limited to an α-oxy C(sp^3^)‒H arylation of ethers. MacMillan and co-workers disclosed a method for the selective direct α-arylation of alcohols **18** using photoredox, HAT, and nickel triple catalysts (Scheme 11) [63]. Here, the use of a zinc-based Lewis acid (LA) was found to activate α-hydroxy C‒H bonds by forming alkoxide (O‒LA) and suppressing the C‒O bond formation by inhibiting the formation of a nickel alkoxide species. The authors also claimed that the use of the zinc-based LA also deactivates the other hydridic bonds such as α-amino and α-oxy C‒H bonds. Among the tested 24 Lewis acids, the zinc salts (ZnCl_2_ and ZnBr_2_) gave the best results. The method's potency was further shown by the synthesis of the drug fluoxetine (**21**) in three steps (Scheme 12) [63]. The transformation was proposed to proceed via a similar mechanism to the one shown in Figure 2.

**Scheme 13:**
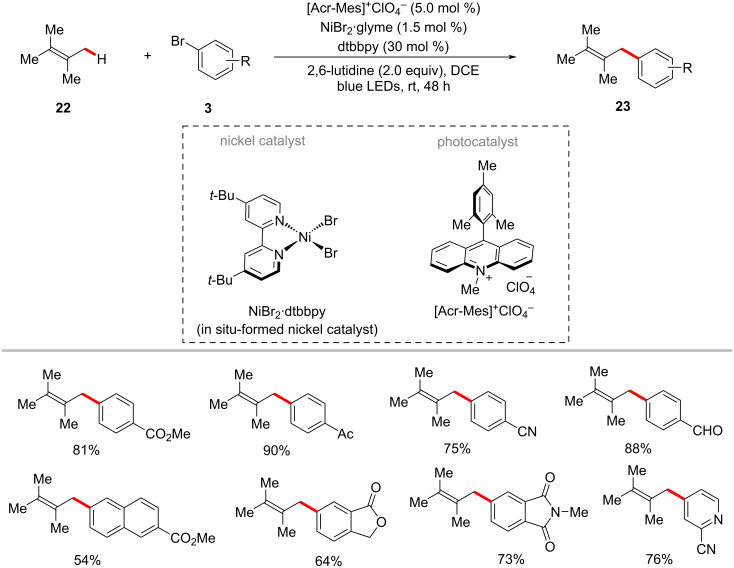
Photochemical nickel-catalyzed allylic C(sp^3^)‒H arylation with aryl bromides.

In 2018, Huang and Rueping devised reaction conditions for the photochemical nickel-catalyzed arylation of allylic C(sp^3^)‒H bonds with aryl bromides **3** in the presence of the organic photocatalyst 9-mesityl-10-methylacridinium perchlorate ([Acr-Mes]^+^ClO_4_^−^) [64]. The reaction was conveniently achieved at room temperature under blue light irradiation. Moreover, as shown in Scheme 13, electron-deficient aryl bromides were efficient in forming the desired products **23** in optimal yields. In contrast, only trace amounts of cross-coupled products were observed when unsubstituted and electron-rich aryl bromides were used.

**Figure 7:**
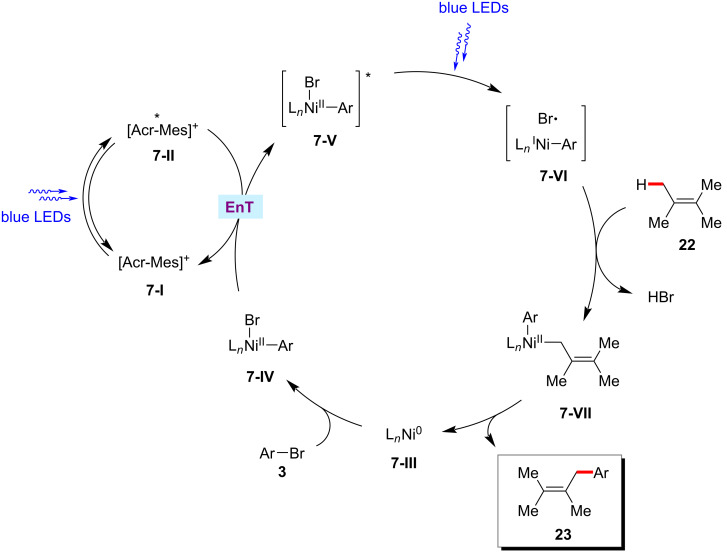
Proposed mechanism for the photochemical nickel-catalyzed allylic C(sp^3^)‒H arylation with aryl bromides.

Based on their experimental results, the authors proposed that a triplet–triplet energy transfer occurs between the nickel(II)–aryl species **7-IV** and the excited acridinium photocatalyst *Mes-Acr-Me^+^ **7-II** (Figure 7) [64]. Homolysis of the excited nickel(II) species **7-V** results in the formation of a bromine radical, which then readily abstracts the allylic C(sp^3^)‒H to give the allylic radical species. Thus, the generated allylic radical species rebound to nickel complex and followed by reductive elimination delivers the desired product **23** and the active nickel(0) species **7-III**.

**Scheme 14:**
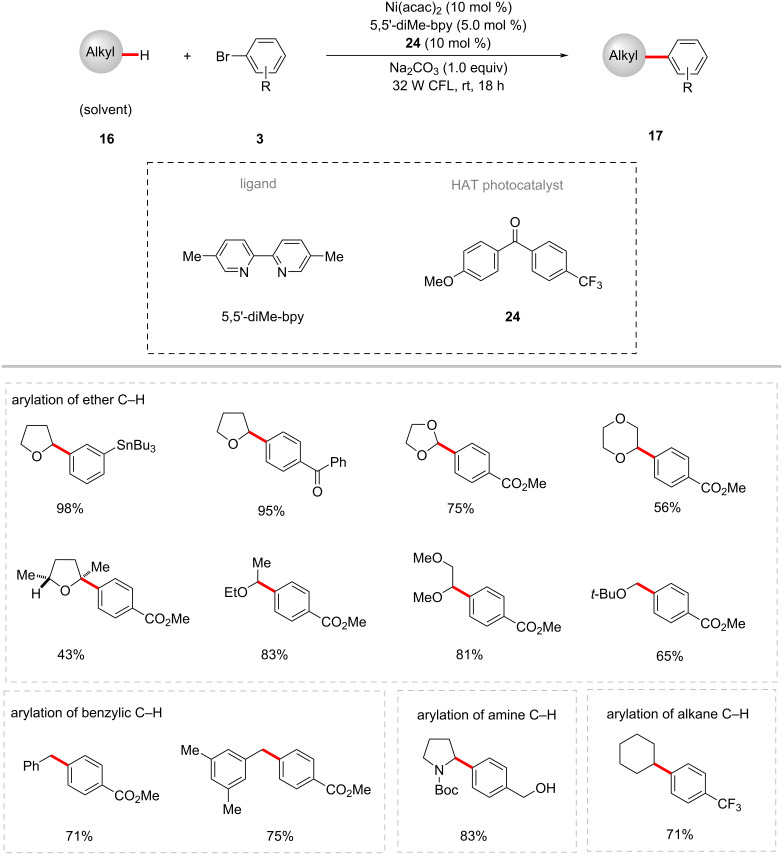
Photochemical C(sp^3^)‒H arylation by the synergy of ketone HAT catalysis and nickel catalysis.

**Figure 8:**
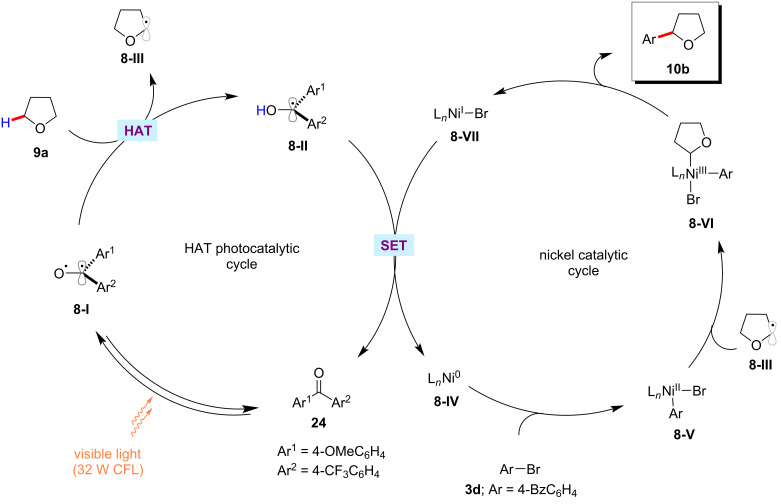
Proposed mechanism for photochemical C(sp^3^)‒H arylation by the synergy of ketone HAT catalysis and nickel catalysis.

The triplet ketone sensitizers can also be employed in the HAT and SET processes [65]. Thus, Martin and co-workers presented an example of the arylation of α-oxy C(sp^3^)‒H bonds of ethers **9** with aryl bromides **3** employing synergy between the nickel catalysis and ketone HAT photocatalyst [66]. Here, the catalytic system composed of the ketone photocatalyst (4-methoxyphenyl)(4-(trifluoromethyl)phenyl)methanone (**24**), Ni(acac)_2_, 5,5’-dimethyl-2,2’-bipyridine (5,5’-diMe-bpy), Na_2_CO_3_ under visible light (CFL) irradiation was found to be optimal to provide the desired arylated products **17** (Scheme 14). Both electron-deficient and electron-rich aryl bromides proved viable substrates and afforded the products **10/17** in good yields. In addition to a variety of cyclic and acyclic ethers, amines, benzylic and alkane C(sp^3^)‒H bonds were also arylated under similar reaction conditions with moderate to good yields. Based on their detailed mechanistic studies, the authors proposed a possible catalytic cycle involving a C–H cleavage via a HAT process between the triplet excited ketone photocatalyst **24** and the C(sp^3^)–H substrates (Figure 8) [66]. Thus, the formed carbon-centered radical species **8-III** combines with the nickel(II)–aryl intermediate **8-V** to form nickel(III) species **8-VI,** which readily undergoes a reductive elimination to deliver the cross-coupled product **10** and nickel(I) species **8-VII**. The SET process between the ketyl radical **8-II** and the nickel(I) species **8-VII** regenerates the active nickel(0) catalytic species **8-IV** and the ketone photocatalyst **24**.

**Scheme 15:**
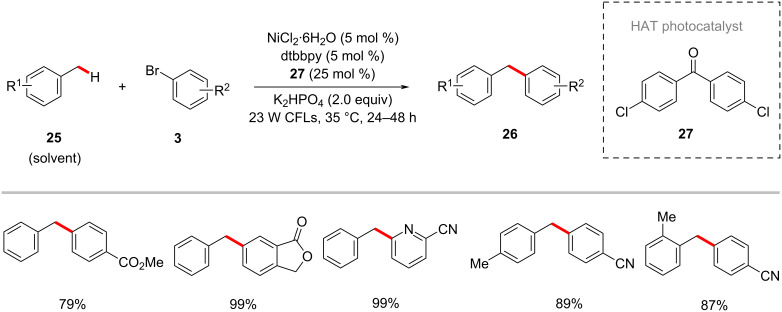
Benzophenone- and nickel-catalyzed photoredox benzylic C–H arylation.

In a related process, Rueping employed 4,4’-dichlorobenzophenone (**27**) as the HAT photocatalyst along with a nickel catalyst for the direct arylation of benzylic C–H bonds with aryl bromides **3** under visible light irradiation at 35 °C (Scheme 15) [67]. Here, the diaryl ketone photocatalyst played a dual role as hydrogen-atom-transfer (HAT) and electron-transfer agent. This C–H arylation protocol provided the diarylmethane derivatives **26** in moderate to good yields.

**Scheme 16:**
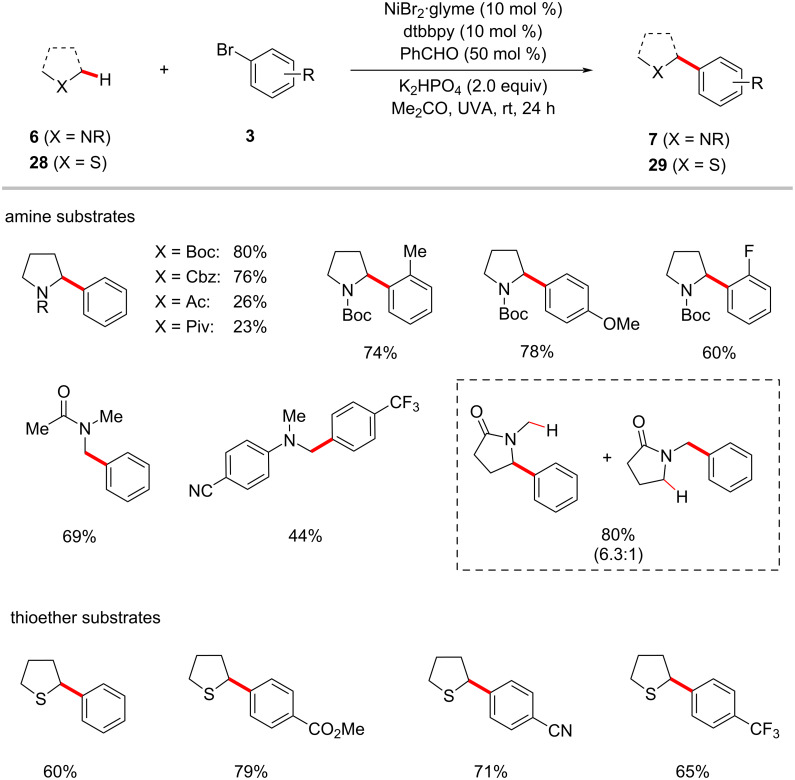
Benzaldehyde- and nickel-catalyzed photoredox C(sp^3^)–H arylation.

In 2019, the Hashmi group discovered the synergistic combination of nickel catalysis and benzaldehyde for the arylation of C(sp^3^)–H bonds adjacent to nitrogen or sulfur in amides **6** and thioethers **28**, respectively, under UVA light irradiation [68]. As shown in Scheme 16, both primary and secondary C(sp^3^)–H bonds of amides were arylated with moderate to good yields. When both primary and secondary C(sp^3^)‒H bonds are present in the substrate, regioselectivity favors the secondary position. The catalytic reaction conditions were compatible with the C(sp^3^)‒H arylation of tetrahydrothiophene (**28a**) as well [68].

**Scheme 17:**
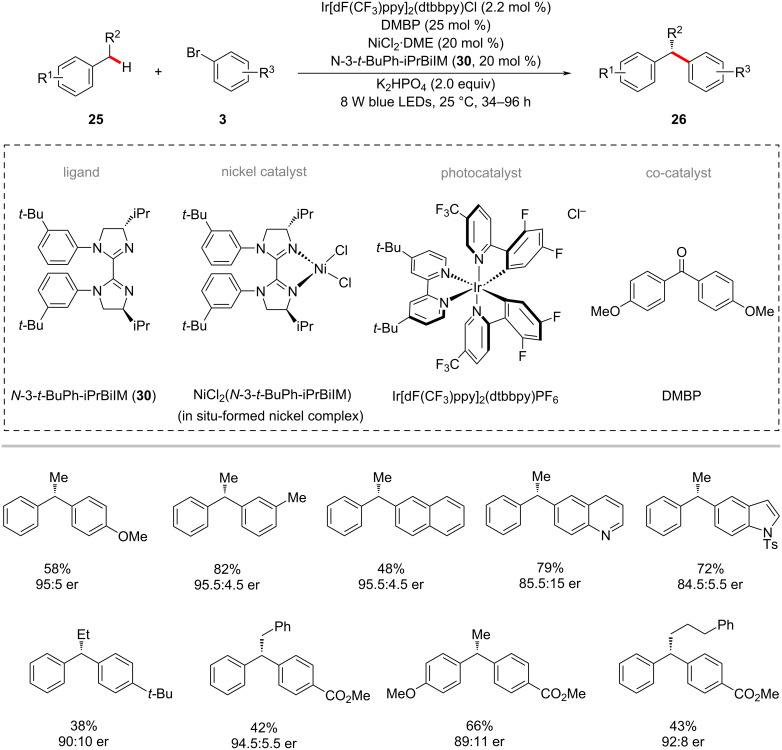
Photoredox and nickel-catalyzed enantioselective benzylic C–H arylation.

**Figure 9:**
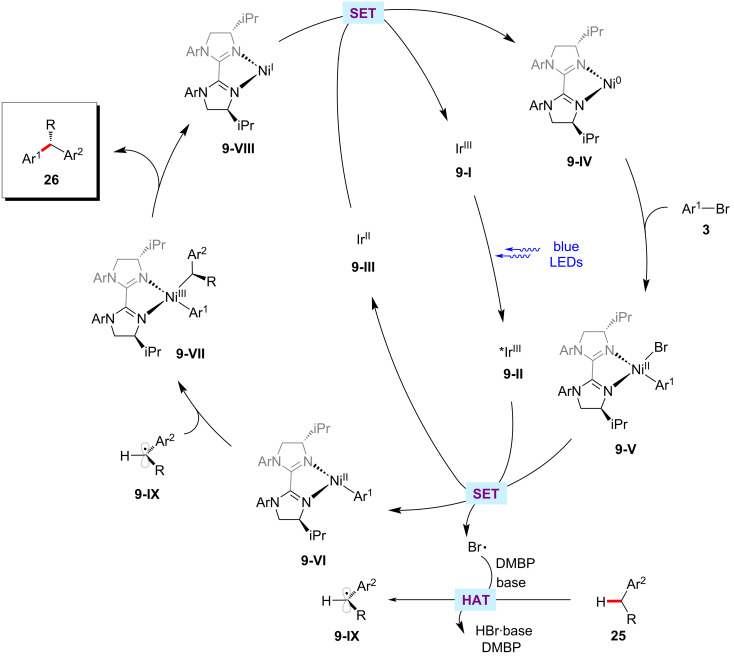
Proposed mechanism for the photoredox and nickel-catalyzed enantioselective benzylic C–H arylation.

The enantioselective C–H functionalization is a valuable method for synthesizing useful organic compounds from simple alkane starting materials [51,69–70]. Recently, Lu and co-workers reported an enantioselective benzylic C–H arylation method for synthesizing 1,1-diarylalkanes **26** via a photoredox and nickel dual catalysis (Scheme 17) [71]. The reaction relied on the chiral biimidazoline ligand **30**, which gave the best results among various tested chiral bioxazolines and chiral biimidazoline ligands. Notably, the aryl substituent at the imidazoline nitrogen of the ligands significantly affected the product yields and enantioselectivities. A wide range of aryl bromides **3** were tested with alkylbenzenes **25** under ambient reaction conditions and afforded the desired products **26** in moderate yields and good enantioselectivities. Based on their control experiments and mechanistic studies, it was postulated that a bromine radical might be involved in the HAT process of benzylic C‒H bond using DMBP as co-catalyst to deliver benzylic radical species **9-IX** (Figure 9) [71]. The benzylic radical **9-IX** intercepted with the nickel catalytic cycle to result in the desired products **26**.

**Scheme 18:**
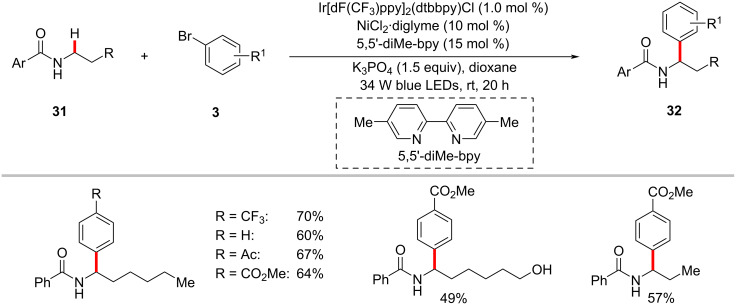
Photoredox nickel-catalyzed α-(sp^3^)‒H arylation of secondary benzamides with aryl bromides.

**Scheme 19:**
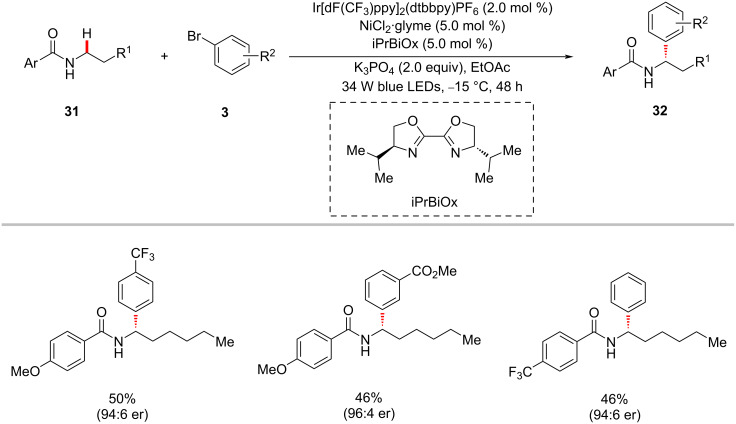
Enantioselective sp^3^ α-arylation of benzamides.

The photoredox nickel-catalyzed arylation of α-amino C(sp^3^)–H bonds are not limited to tertiary amines/amides. Secondary amides could also be arylated, as reported by Montgomery, Martin and co-workers [72]. The authors discovered that the combination of Ir[dF(CF_3_)ppy]_2_(dtbbpy)PF_6_, NiBr_2_·diglyme, 5,5’-dimethyl-2,2’-bipyridine (5,5’-diMe-bpy), and K_3_PO_4_ in dioxane under irradiation of blue LEDs at ambient temperature afforded the desired α-arylation products **32** from secondary amides **31** and (hetero)aryl bromides **3** (Scheme 18) [72]. The method showed a broad substrate scope for both amides and aryl bromides. The authors also realized the enantioselective variant of this protocol using the chiral iPrBiOx ligand under slightly modified reaction conditions (Scheme 19) [72].

**Scheme 20:**
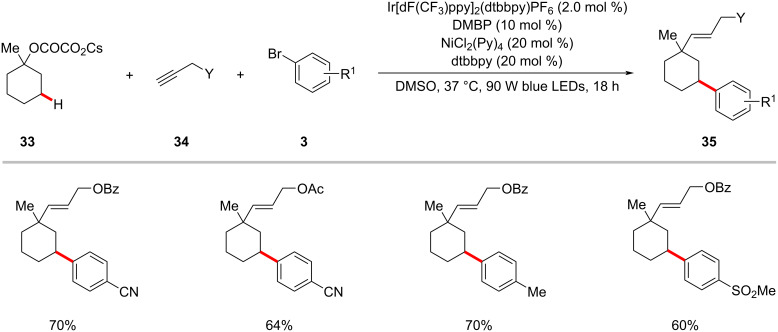
Nickel-catalyzed decarboxylative vinylation/C‒H arylation of cyclic oxalates.

**Figure 10:**
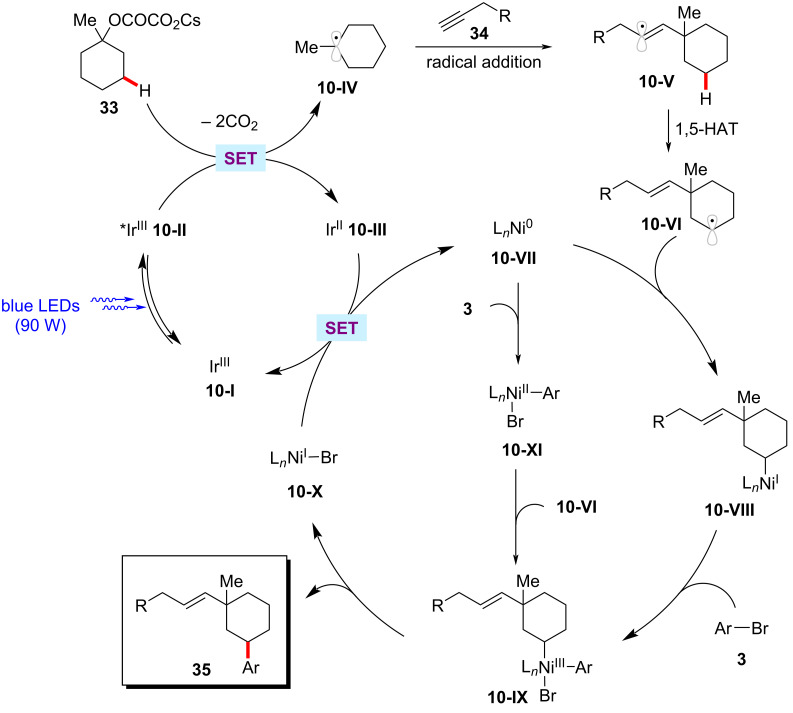
Proposed mechanism for the nickel-catalyzed decarboxylative vinylation/C‒H arylation of cyclic oxalates.

Recently, Chu achieved the selective assembly of vinyl and aryl functionalities onto saturated cyclic hydrocarbons via a photoredox nickel-catalyzed sequential C–O decarboxylative vinylation/arylation of cyclic oxalates **33** with terminal alkyne **34** and aryl bromides **3** (Scheme 20) [73]. As to the scope, aryl bromides **3** containing various electron-withdrawing substituents displayed better efficiency over the electron-rich aryl bromides. The authors proposed a plausible catalytic cycle to account for the mode of action of this cascade arylation protocol (Figure 10) [73]. In the photocatalytic cycle, the SET event between the photoexcited iridium catalyst **10-II** and the substrate oxalate **33** generates a tertiary carbon-centered radical **10-IV** by decarboxylation and the reduced iridium(II) photocatalyst **10-III**. The active iridium(III) photocatalyst **10-I** is regenerated by a SET process between **10-III** and the nickel(I) species **10-X**. The addition of the tertiary radical **10-IV** to the terminal alkyne **34** followed by an intramolecular 1,5-HAT results in a nucleophilic secondary alkyl radical species **10-VI**. Subsequently, the alkyl radical **10-VI** intercepts nickel(0) complex **10-VII** to form a nickel(I)–alkyl intermediate **10-VIII**, which then undergoes oxidative addition to aryl bromide **3** followed by reductive elimination furnishing the desired product **35** and the nickel(I) species **10-X**. The authors noted that the oxidative addition of the nickel(0) species **10-VII** to aryl bromide **3** and subsequent steps to produce nickel(III) intermediate **10-IX** could not be ruled out.

**Scheme 21:**
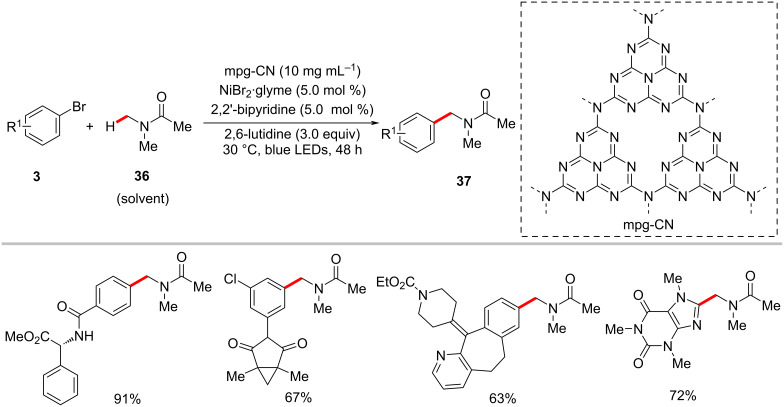
C(sp^3^)−H arylation of bioactive molecules using mpg-CN photocatalysis and nickel catalysis.

**Figure 11:**
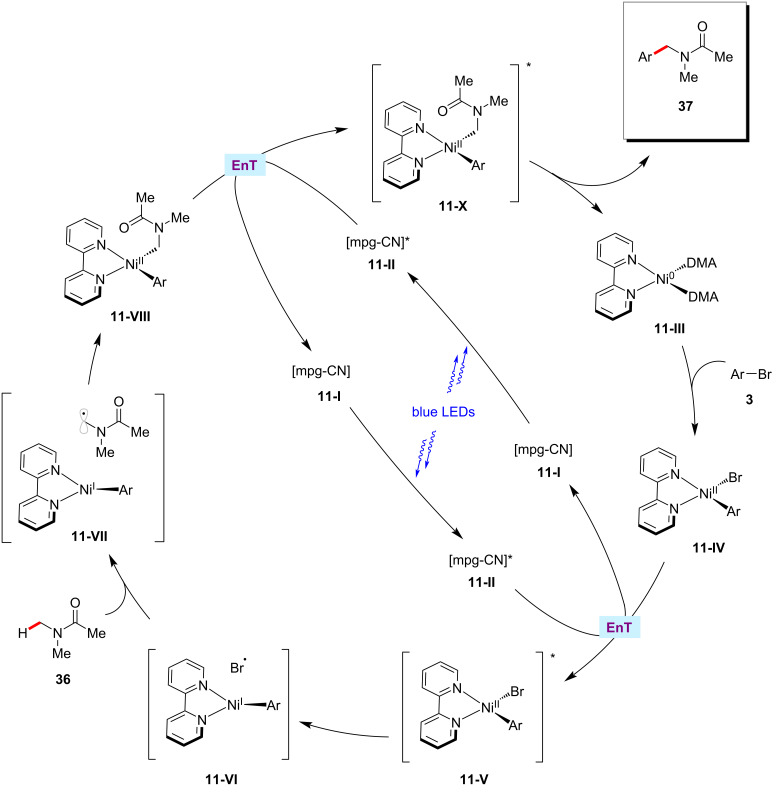
Proposed mechanism for the mpg-CN/nickel photocatalytic C(sp^3^)–H arylation.

The König group discovered that the arylation of α-amino C(sp^3^)–H bonds could be realized with aryl halides using mesoporous graphitic carbon nitride (mpg-CN) [74–76] as a heterogeneous organic semiconductor photocatalyst in combination with nickel catalysis [77]. Here, the catalytic system consisting of NiBr_2_·glyme, 2,2′-bipyridine, 2,6-lutidine, and mpg-CN under blue light irradiation at ambient temperature was found to be optimal to furnish the desired cross-coupled products **37** in satisfactory yields. Furthermore, the method proved applicable to the late-stage diversiﬁcation of bioactive molecules, pharmaceuticals, and agrochemicals as aryl coupling partners (Scheme 21) [77]. The authors proposed a catalytic cycle that involves an energy-transfer pathway generating an electronically excited nickel complex as a key reactive intermediate (Figure 11).

**Scheme 22:**
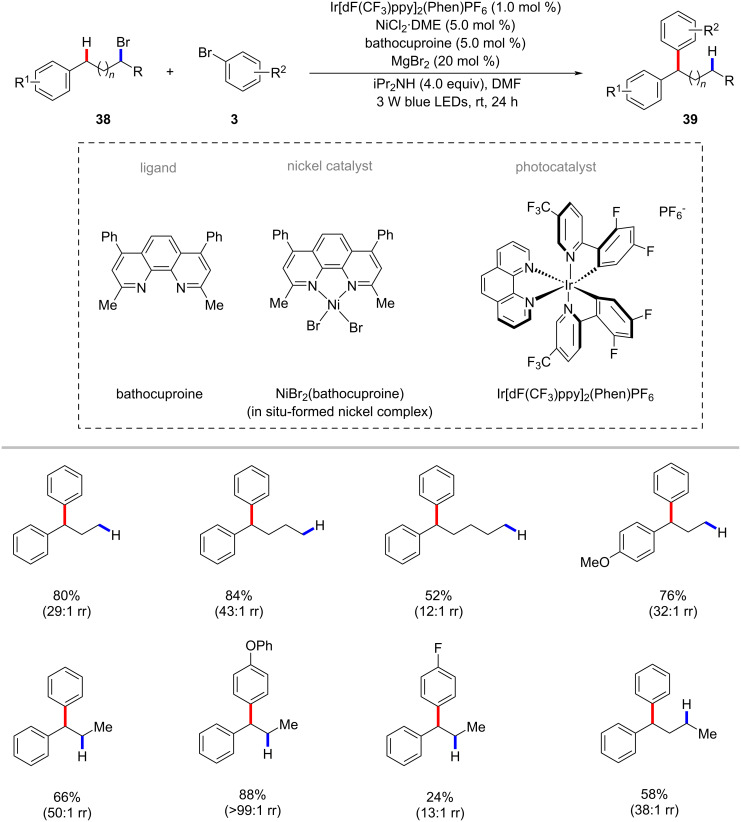
Nickel-catalyzed synthesis of 1,1-diarylalkanes from alkyl bromides and aryl bromides.

Photochemical nickel catalysis was used to synthesize 1,1-diarylalkanes **39** from unactivated alkyl bromides **38** and aryl bromides **3** through a reductive migratory cross-coupling strategy (Scheme 22) [78]. The use of an iridium-based photocatalyst along with stoichiometric diisopropylamine as the terminal reductant were found to be beneficial to obtain the desired products **39**. Both primary and secondary alkyl bromides **38** proved viable substrates to give the benzylic arylation products **39** with good regioselectivities. The authors proposed a tentative visible-light-driven radical chain mechanistic profile with nickel chain-walking as a key step to rationalize the C–H arylation process [78].

### Alkylation

**Figure 12:**
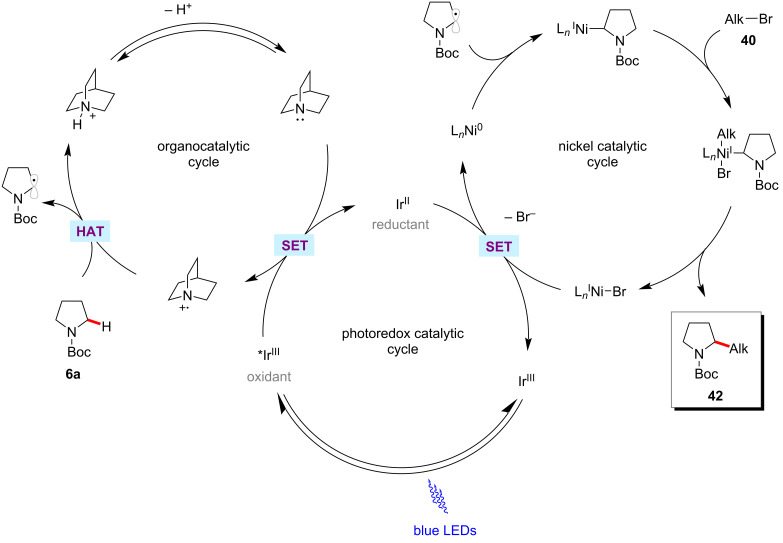
Proposed mechanism for photoredox nickel-catalyzed C(sp^3^)–H alkylation via polarity-matched HAT.

**Scheme 23:**
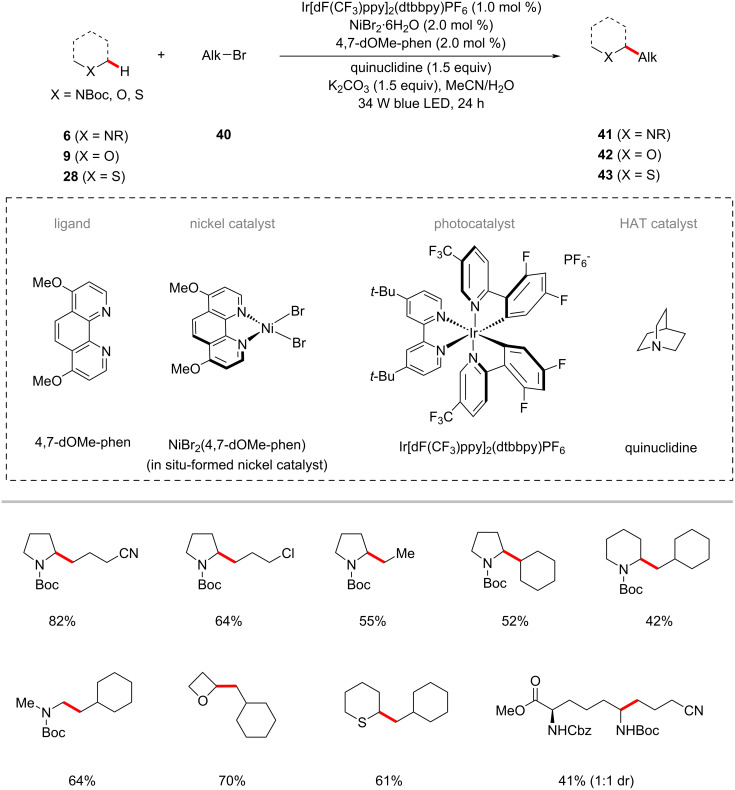
Photoredox nickel-catalyzed C(sp^3^)‒H alkylation via polarity-matched HAT.

The direct functionalization of C–H bonds in alkyl groups is a fundamental but challenging operation in organic synthesis. While significant advances had been accomplished with (hetero)aromatic C(sp^2^)–H alkylations [79–81], examples for C(sp^3^)–C(sp^3^) couplings through C–H activation are scarce [82–84]. In this context, a synergistic combination of photoredox catalysis and nickel catalysis is also often employed to C(sp^3^)‒H alkylation transformations. For example, in 2017, MacMillan and co-workers reported a selective C(sp^3^)–H alkylation protocol via polarity-matched hydrogen atom transfer (HAT) using photoredox and nickel catalysis [85]. This method works through synergistic cooperation of three catalytic cycles of photoredox, nickel, and HAT catalysis (Figure 12). The HAT-metallaphotoredox process selectively alkylates α-C–H of amines **6**, ethers **9**, and sulfides **28** with a variety of alkyl bromides **40** (Scheme 23).

**Scheme 24:**
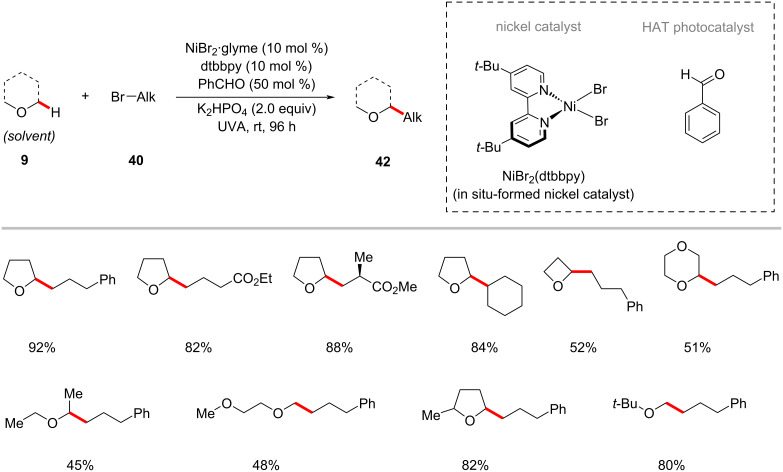
Benzaldehyde- and nickel-catalyzed photoredox C(sp^3^)‒H alkylation of ethers.

**Scheme 25:**
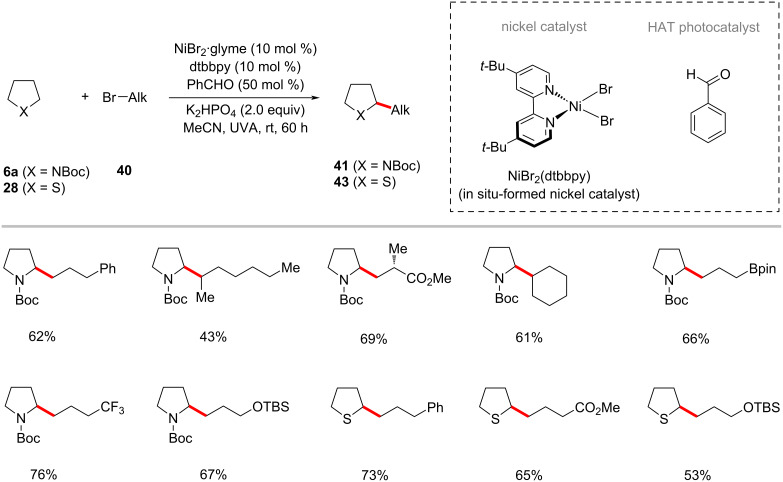
Benzaldehyde- and nickel-catalyzed photoredox C(sp^3^)‒H alkylation of amides and thioethers.

The Hashmi group further developed the photoredox nickel-catalyzed C–H alkylation strategy to use the readily available inexpensive organo-photocatalyst benzaldehyde as the HAT photocatalyst under UVA irradiation [68,86]. Thus, the combination of NiBr_2_·glyme/dtbbpy, benzaldehyde as both the photosensitizer and hydrogen abstractor, and K_2_HPO_4_ as a base under irradiation with UVA light enabled the cross-coupling of α-oxy C–H bonds of acyclic/cyclic ethers **9** with alkyl bromides **40** (Scheme 24) [86]. The catalytic system was not limited to α-oxy C–H bonds of cyclic ethers, substrates having other heteroatoms such as nitrogen and sulfur that can imbue a hydridic nature of their α-C–H also proved viable under slightly modiﬁed reaction conditions, as was reported by Hashmi in 2019 (Scheme 25) [68].

**Scheme 26:**
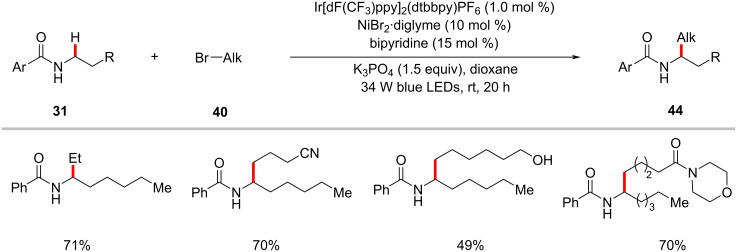
Photoredox and nickel-catalyzed C(sp^3^)‒H alkylation of benzamides with alkyl bromides.

In a recent publication, the group of Martin enabled an intermolecular alkylation of α-amino C–H bonds of benzamides **31** with unactivated alkyl halides **40** [72]. In this transformation, the combination of NiBr_2_·diglyme/bipyridine in the presence of the iridium photocatalyst Ir[dF(CF_3_)ppy]_2_(dtbbpy)PF_6_ under blue light irradiation was found to be appropriate to give optimal results (Scheme 26) [72].

**Scheme 27:**
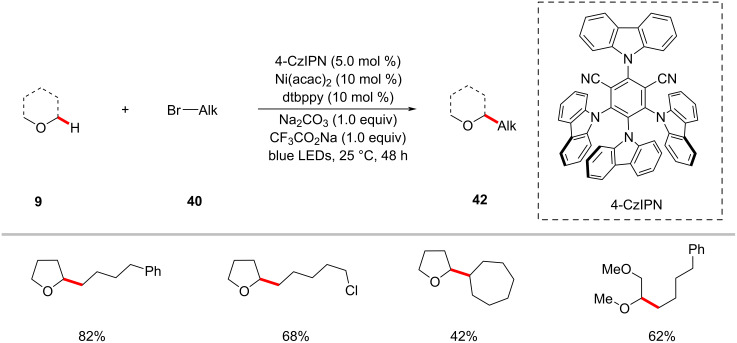
CzIPN and nickel-catalyzed C(sp^3^)‒H alkylation of ethers with alkyl bromides.

**Figure 13:**
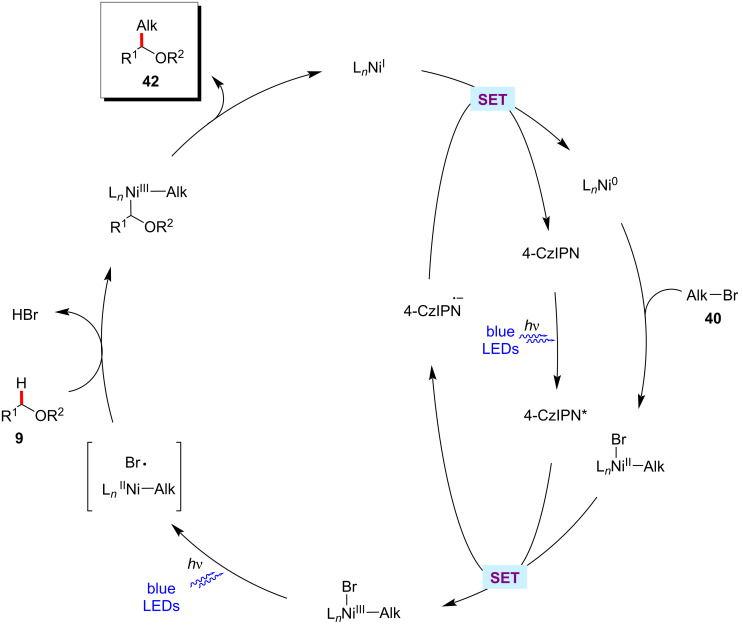
Proposed mechanism for the CzIPN and nickel-catalyzed C(sp^3^)‒H alkylation of ethers.

The König group recently disclosed that the organic photocatalyst 1,2,3,5- tetrakis(carbazol-9-yl)-4,6-dicyanobenzene (4-CzIPN) could also be used with nickel catalysis for the alkylation of α-oxy C–H bonds of acyclic/cyclic ethers **9** with alkyl halides **40** (Scheme 27) [87]. The bench stable nickel(II) acetylacetonate can be used as the catalyst along with the dtbbpy ligand. The authors proposed a plausible reaction mechanism to account for the mode of operation as shown in Figure 13 [87]. Here, the halide radical species generated in situ was proposed to mediate the HAT event.

**Scheme 28:**
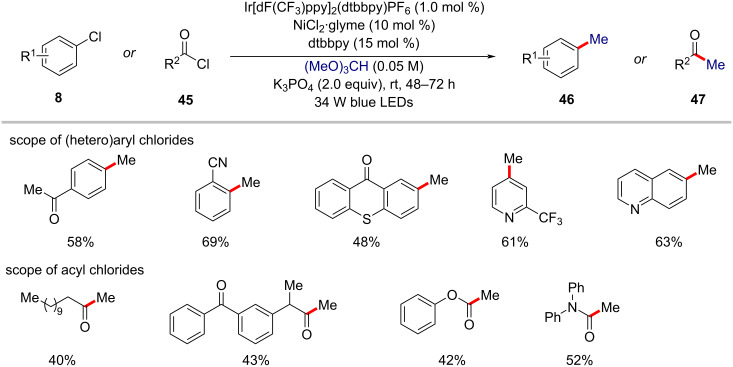
Nickel/photoredox-catalyzed methylation of (hetero)aryl chlorides and acid chlorides using trimethyl orthoformate.

**Figure 14:**
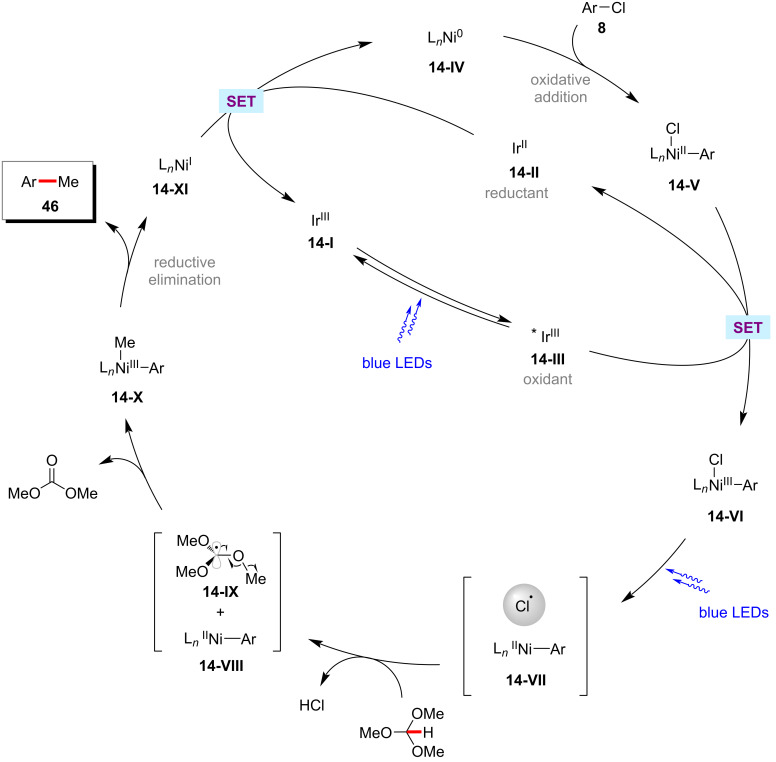
Proposed catalytic cycle for the nickel/photoredox-catalyzed methylation of (hetero)aryl chlorides using trimethyl orthoformate.

Considering the 'magic methyl' effect in drug candidates [88], there is a strong demand for the direct methylation of C–H bonds because it would provide a convenient access to structures that might not otherwise be available for biological testing [89–91]. Hence, Doyle and co-workers realized an elegant approach for the methylation of (hetero)aryl chlorides **8** using trimethyl orthoformate as a methyl radical source via a nickel/photoredox-catalyzed HAT processes (Scheme 28) [92]. The method was also compatible with other chlorine-containing electrophiles such as acyl chlorides **45** to afford methyl ketones **47** in moderate yields. Based on the detailed mechanistic studies, the authors proposed a catalytic cycle involving the generation of methyl radicals via β-scission of a tertiary radical which in turn was generated from trimethyl orthoformate by a photogenerated chlorine radical-mediated HAT process (Figure 14) [92].

**Scheme 29:**
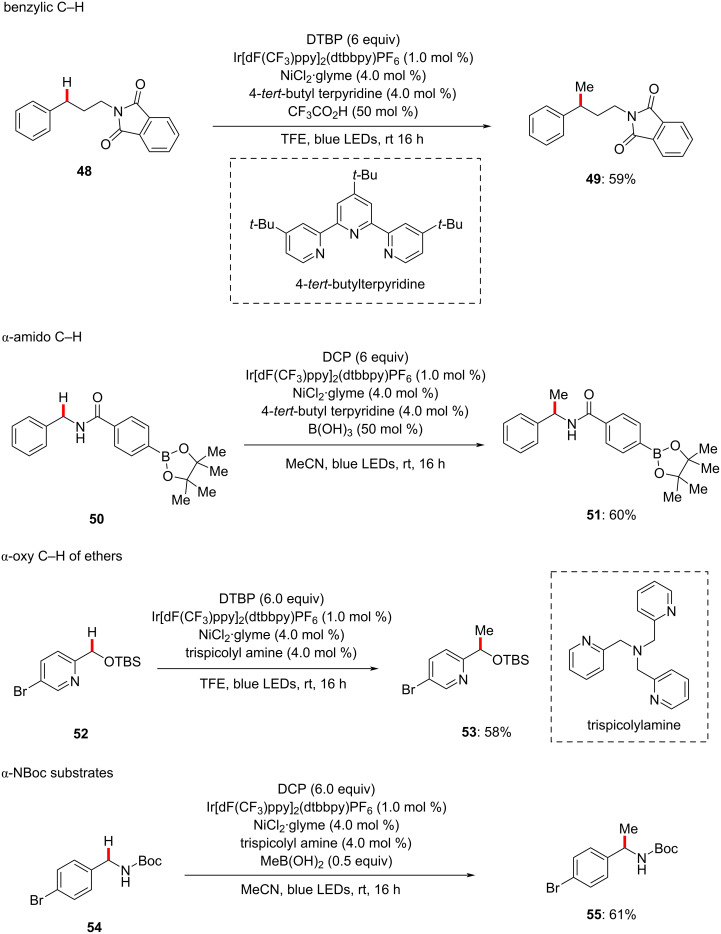
Photochemical nickel-catalyzed C(sp^3^)–H methylations.

Recently, Stahl devised a photoredox nickel-catalyzed methylation of benzylic and α-amino C(sp^3^)–H bonds using di-*tert*-butyl peroxide (DTBP) or dicumyl peroxide (DCP) as the methyl source under mild conditions [93]. Based on the substrate structure and peroxide choice, the authors developed four sets of reaction conditions (Scheme 29) [93]. In these reaction conditions, photocatalyst Ir[dF(CF_3_)ppy]_2_(dtbbpy)PF_6_ and nickel catalyst NiCl_2_·glyme were identified to be optimal and common.

**Scheme 30:**
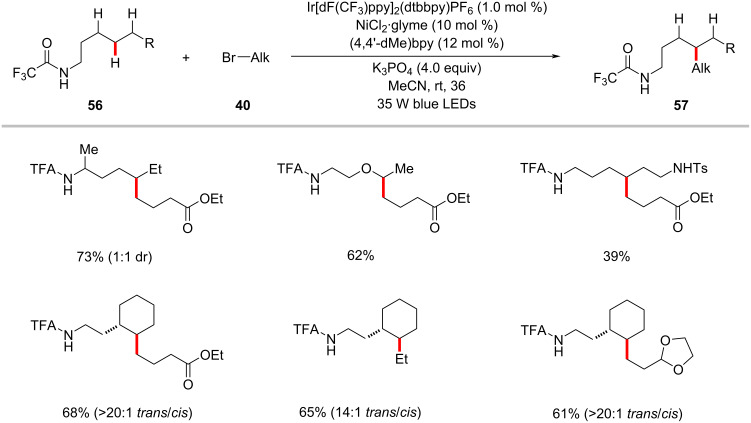
Photoredox nickel catalysis-enabled alkylation of unactivated C(sp^3^)–H bonds with alkyl bromides.

The nickel-catalyzed photoredox-enabled HAT strategy was exploited for the remote functionalization of C(sp^3^)–H with alkyl halides as was disclosed by Rovis and co-workers [94]. Thus, a variety of linear amides were alkylated selectively at the δ-methylene position through an intramolecular 1,5-HAT event in synergy with a nickel catalytic cycle. Interestingly, secondary C–H bonds are selectively functionalized in preference over primary C–H, in the case of multiple functionalizable sites were available. The authors examined ample scope of alkyl trifluoroacetamides **56** and alkyl bromides **40** to afford the corresponding alkylated products **57** in moderate to good yields (Scheme 30) [94]. As to the modus operandi, the generation of an alkyl radical species through amide directed 1,5-HAT followed by capture of the thus formed alkyl radical by the nickel catalyst was proposed.

### Alkenylation

**Scheme 31:**
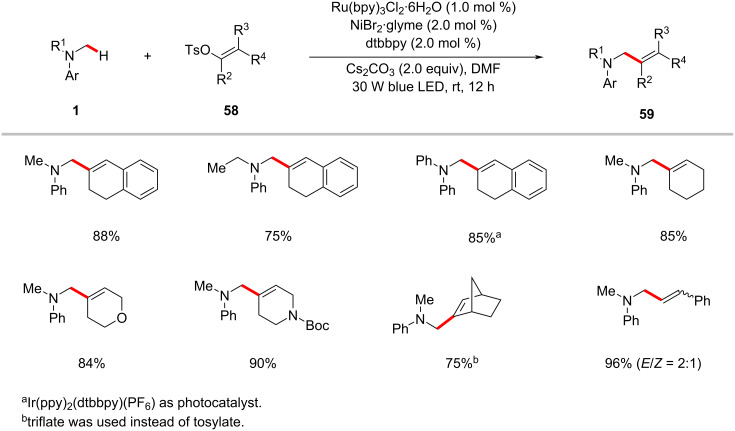
Photochemical C(sp^3^)–H alkenylation with alkenyl tosylates.

Over the past few decades, outstanding progress has been realized in the direct alkenylation transformation of C(sp^2^)–H bonds [95–101]. However, the related C(sp^3^)–H alkenylation is much less developed due to lower reactivity, poor regioselectivities and the need of noble metal catalysts [50,102–106]. Recently, Yu and co-workers conveniently achieved the direct alkenylation of α-amino C(sp^3^)–H bonds of amines **1** with alkenyl tosylates **58**. The combination of the Ru(bpy)_3_Cl_2_·6H_2_O photocatalyst and NiCl_2_·glyme as the nickel catalyst enabled this C–H alkenylation protocol using alkenyl C(sp^2^)–O electrophiles at ambient reaction temperature under blue light irradiation (Scheme 31) [58]. In general, the method displayed broad substrate scope, good functional group tolerance, and excellent regioselectivities.

**Scheme 32:**
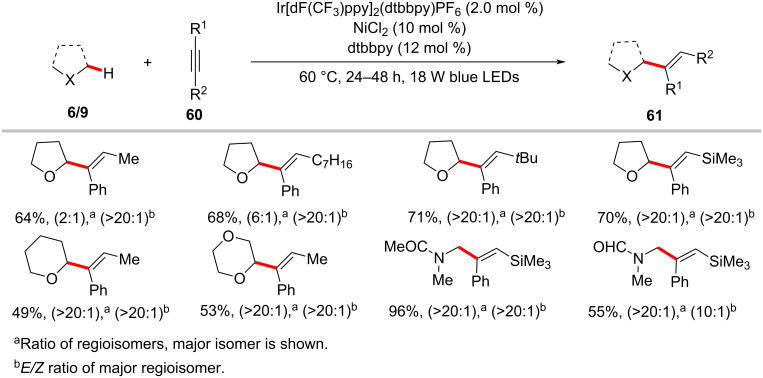
Photoredox nickel-catalyzed hydroalkylation of internal alkynes.

**Figure 15:**
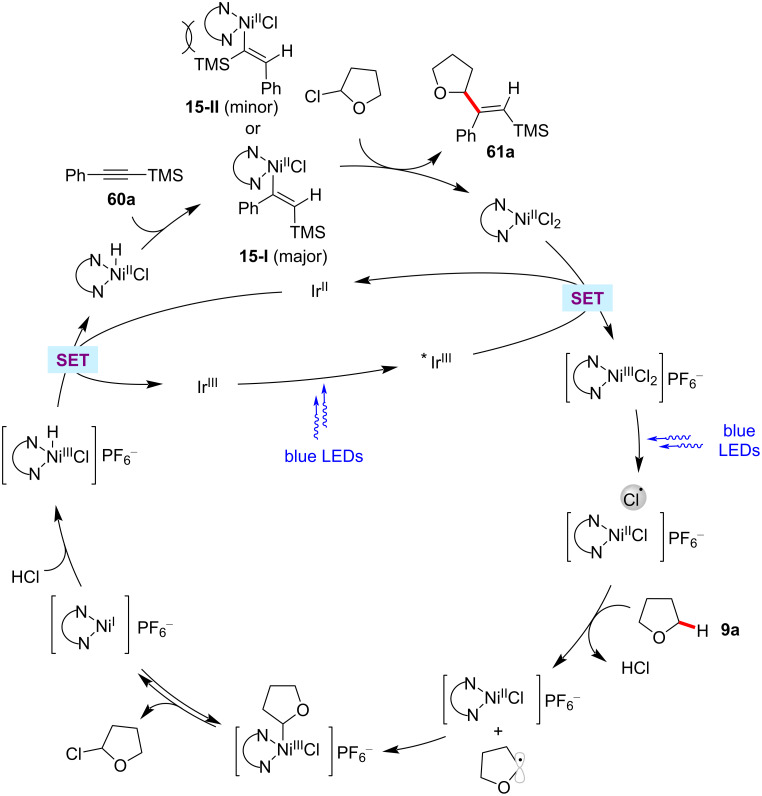
Proposed mechanism for the photoredox nickel-catalyzed hydroalkylation of internal alkynes.

In 2017, the Wu group reported a notable C(sp^3^)–H functionalization process with internal alkynes by means of photoredox nickel catalysis [107]. Within this study, they showed that the reaction of ethers, or amides with internal alkynes **60** in the presence of the combination of a catalytic amount of Ir[dF(CF_3_)ppy]_2_(dtbbpy)PF_6_, NiCl_2_, and dtbbpy as ligand at 60 °C under blue LED light irradiation gave alkenylation products **61** in good yields (Scheme 32) [107]. In general, the reaction proceeded with good regioselectivities and excellent *E*/*Z* ratios. Further, the authors also conducted this process in a continuous-flow reactor. The mechanistic studies indicated that a nickel hydride intermediate generated with C(sp^3^)–H as the hydride source is involved in this catalytic transformation. The hydronickelation step results in the sterically less hindered vinylnickel intermediate **15-I**, which corresponds to the observed major isomer product (Figure 15).

**Scheme 33:**
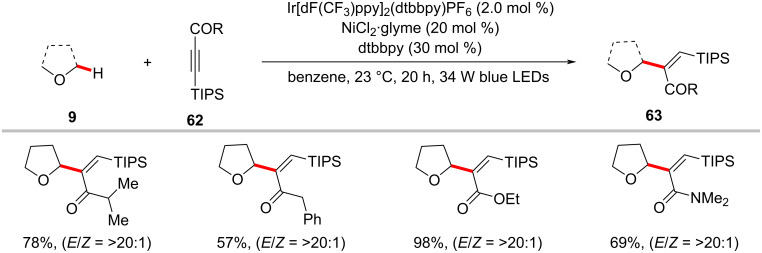
Photoredox nickel-catalyzed hydroalkylation of activated alkynes with C(sp^3^)−H bonds.

In a related transformation, Hong realized the exclusively α-selective hydroacylation of ynones, ynoates, and ynamides via photoredox nickel catalysis. Thus, the combination of nickel and iridium catalysts efficiently catalyzed the regioselective α-C(sp^3^)–H addition of ethers **9** to triisopropylsilyl (TIPS)-substituted alkynes **62** (Scheme 33) [108]. Notably, among the tested nickel salts, NiCl_2_·glyme gave superior outcomes than other nickel(II) salts or nickel(0) catalysts, indicating the essential role of chlorine. As to the scope of the reaction, TIPS-protected ynones, ynoates, and ynamides smoothly transformed into the corresponding trisubstituted alkenes **63** in high regio- and stereoselectivities. A possible mechanism was proposed similar to the one shown in Figure 15 to account for the observed high regioselectivity.

### Allylation

**Scheme 34:**
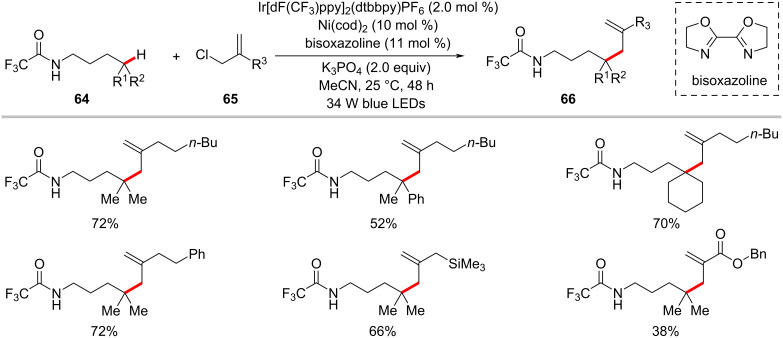
Allylation of unactivated C(sp^3^)−H bonds with allylic chlorides.

The transition-metal-catalyzed direct allylation of unactivated C–H bonds is considered as the prevalent strategy in organic synthesis. Despite significant advances were accomplished in the allylation of (hetero)aromatic and alkenyl C(sp^2^)‒H bonds [109], related reactions of C(sp^3^)–H are less explored [110–111]. In this context, Tambar developed a δ-selective C(sp^3^)–H allylation of aliphatic amides **64** using allyl chlorides **65** under visible light photoredox nickel catalysis (Scheme 34) [112]. The optimized reaction conditions exhibited good tolerance to a variety of substitutions on the allyl chloride substrates **65** and the amide substrates **64**. However, the role of the nickel catalyst in this process and the reaction mechanism pathway were not fully established.

**Scheme 35:**
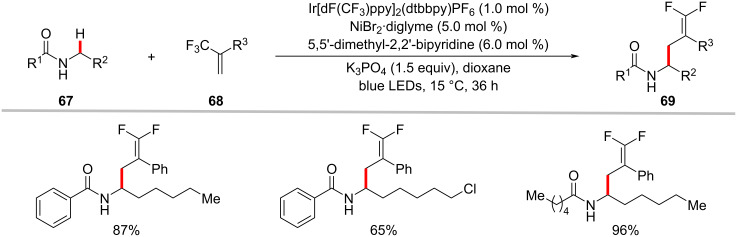
Photochemical nickel-catalyzed α-amino C(sp^3^)–H allylation of secondary amides with trifluoromethylated alkenes.

**Scheme 36:**
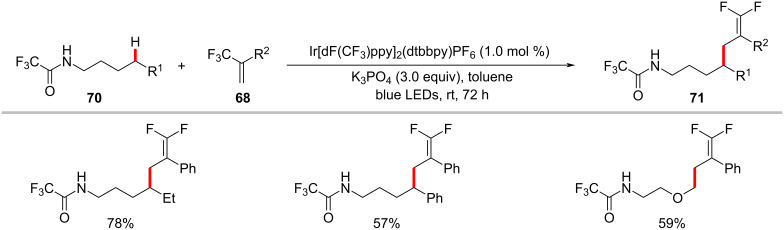
Photoredox δ C(sp^3^)‒H allylation of secondary amides with trifluoromethylated alkenes.

The photoredox nickel-catalyzed allylation of α-amino C(sp^3^)–H bonds with trifluoromethylated alkenes **68** has been more recently achieved by Martin and co-workers (Scheme 35) [113]. This defluorinative functionalization protocol set the stage for the introduction of *gem*-difluoroalkene motifs into α-amino C(sp^3^)–H sites. Interestingly, substrates having a trifluoromethyl group on the amide backbone enabled the functionalization of δ C(sp^3^)–H bonds under slightly modified reaction conditions with exclusion of the nickel catalyst (Scheme 36) [113].

### Acylation

**Scheme 37:**
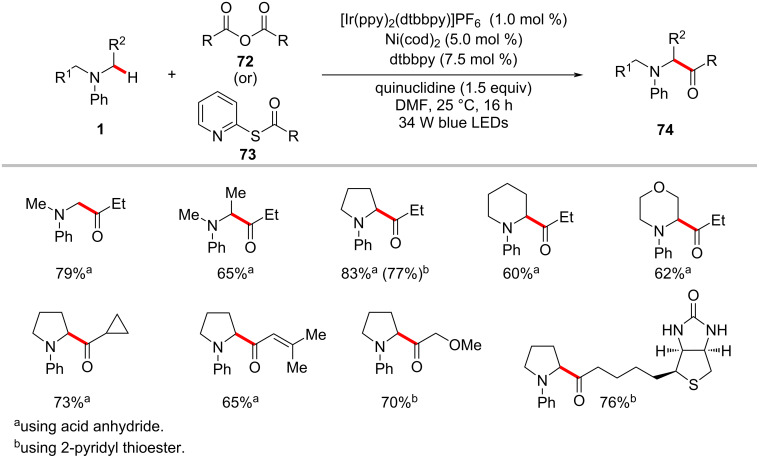
Photoredox nickel-catalyzed acylation of α-amino C(sp^3^)‒H bonds of *N*-arylamines.

**Figure 16:**
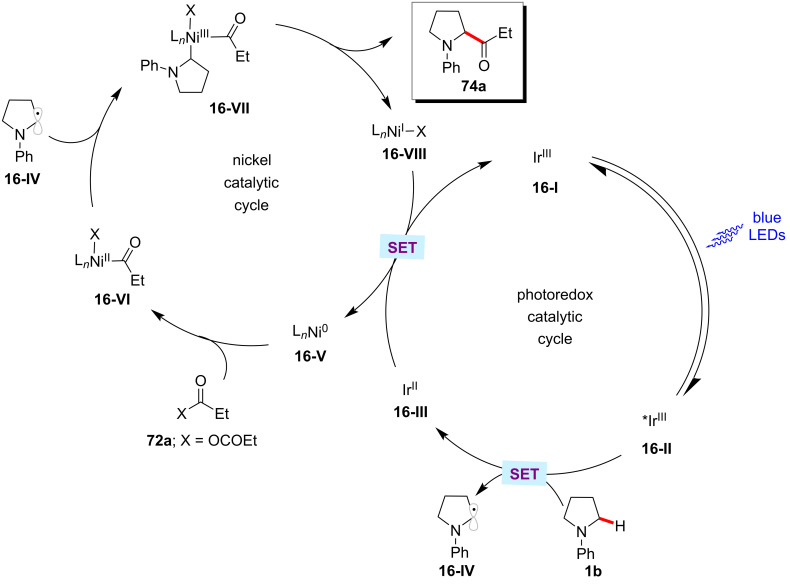
Proposed mechanism for the photoredox nickel-catalyzed acylation of α-amino C(sp^3^)–H bonds of *N*-arylamines.

The ketone motif is an important functional group in pharmaceuticals, agrochemicals, and functional materials [114–117]. Hence continuous efforts devoted to developing a convenient method to introduce keto functional groups onto complex organic molecules. During the last decade, the acylation of hydrocarbons through direct C–H activation has been achieved by means of transition-metal catalysis using various acyl precursors [118–119]. The renaissance of metallaphotoredox catalysis has improved further the C–H acylation procedures by working under mild reaction conditions. Thus, Doyle and Joe reported a mild C–H acylation protocol for the direct functionalization of α-amino C(sp^3^)–H bonds of *N*-arylamines **1** with acyl electrophiles such as anhydrides **72** and 2-pyridyl thioester **73** (Scheme 37) [120]. Here, the combination of the iridium photocatalyst, [Ir(ppy)_2_(dtbbpy)]PF_6_ and Ni(cod)_2_ as the nickel catalyst were found to be optimal to give the desired acylation products **74** in satisfactory yields. Furthermore, a plausible catalytic cycle was proposed to account for the C–H acylation reaction (Figure 16) [120]. A photogenerated α-amino radical **16-IV** intercepts with the nickel catalytic cycle to generate a key nickel(III) intermediate **16-VII**, which readily undergoes reductive elimination to afford the desired cross-coupled product **74a**.

**Scheme 38:**
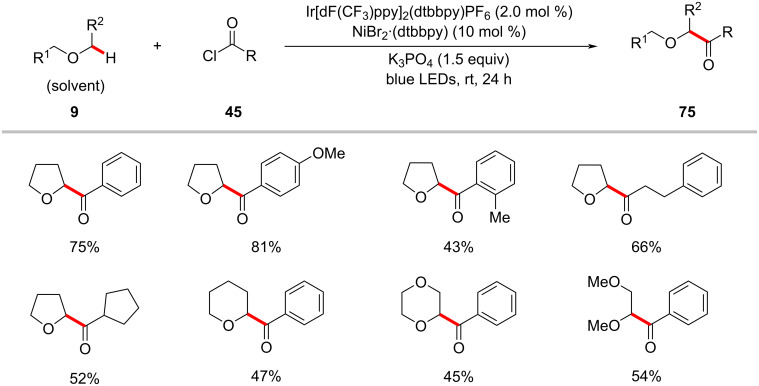
Photocatalytic α‑acylation of ethers with acid chlorides.

**Figure 17:**
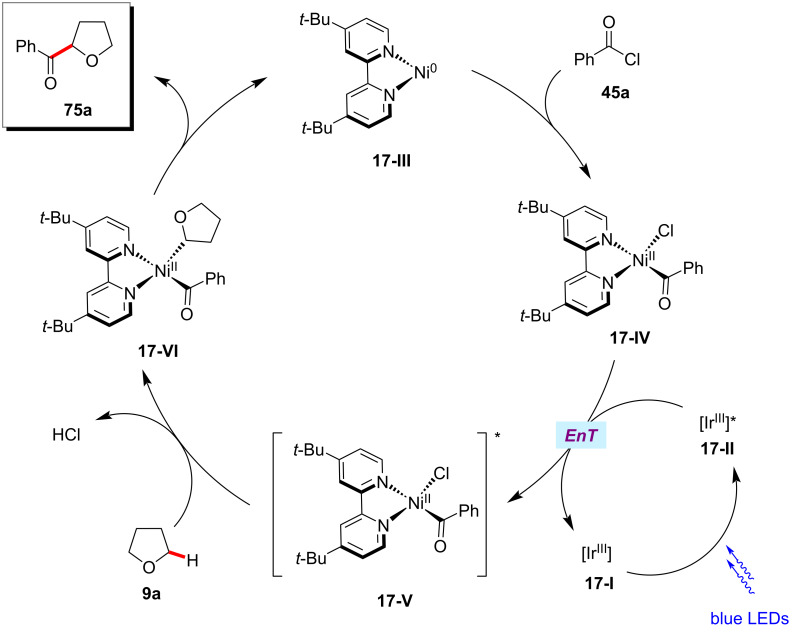
Proposed mechanism for the photocatalytic α‑acylation of ethers with acid chlorides.

In 2017, Kamagai and Shibasaki showed that a robust iridium photocatalysis/nickel catalysis enabled the α-C(sp^3^)–H acylation of ethers **9** with acid chlorides **45** (Scheme 38) [121]. The optimized catalytic conditions were not limited to acid chlorides as acyl sources, and an acid anhydride proved as viable substrate, albeit in a somewhat lower yield. Based on the mechanistic studies, the authors proposed a catalytic cycle involving a triplet–triplet energy transfer between the excited iridium photocatalyst **17-II** and nickel(II) complex **17-IV** (Figure 17) [121]. The excited nickel(II) complex **17-V** undergoes Ni‒Cl bond homolysis followed by a HAT event of the chlorine radical with the ether substrate and subsequent capture of the thus-formed α-oxy C(sp^3^) radical by the nickel complex resulting in the nickel(II)(alkyl)acyl complex **17-VI**. Finally, reductive elimination of **17**-**VI** delivered the desired product **75a**.

**Scheme 39:**
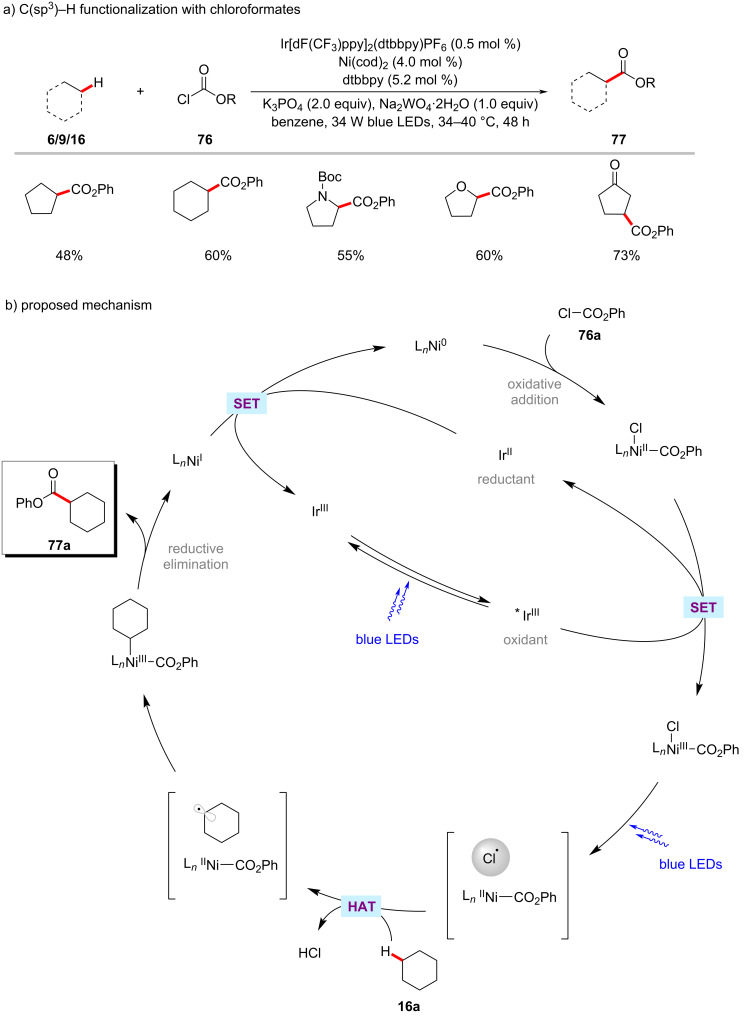
Photoredox and nickel-catalyzed C(sp^3^)‒H esterification with chloroformates.

The nickel-photoredox catalysis was extended to include chloroformates **76** as electrophiles in the C‒H functionalization reaction as was reported by the Doyle group (Scheme 39a) [122]. Here, the combination of Ir[dF(CF_3_)ppy]_2_(dtbbpy)PF_6_ and Ni(cod)_2_ enabled this transformation to proceed under blue light irradiation. Notably, addition of stoichiometric quantities of sodium tungstate were found to be beneficial for the formation of the desired cross-coupling products **77**. The authors’ investigations suggested that tungstate is acting as a base rather than a photocatalyst. A variety of C–H substrates including unactivated alkanes, amines, and ethers were transformed into ester products **77**. A catalytic cycle was proposed with a chlorine radical involved in the HAT event (Scheme 39b) [122].

**Scheme 40:**
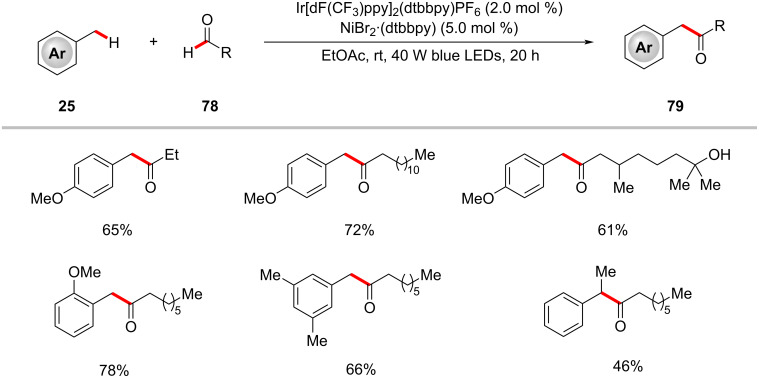
Photoredox nickel-catalyzed dehydrogenative coupling of benzylic and aldehydic C–H bonds.

**Figure 18:**
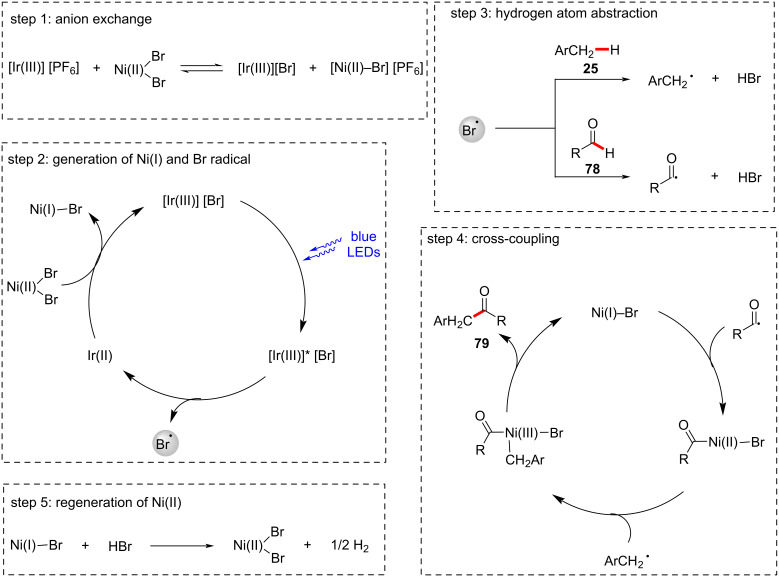
Proposed reaction pathway for the photoredox nickel-catalyzed dehydrogenative coupling of benzylic and aldehydic C–H bonds.

The cooperative activity of an iridium photocatalyst and nickel catalyst also enabled the dehydrogenative cross coupling of benzylic and aldehydic C–H bonds (Scheme 40) [123]. Notably, this method proceeds through a unique mechanism (Figure 18) involving five steps: i) anion exchange between the iridium catalyst and nickel catalyst; ii) generation of a bromine radical and nickel(I) species in the photocatalytic cycle; iii) hydrogen atom abstraction events between the bromine radical and toluene as well as aldehyde; iv) product formation in a nickel catalytic cycle; and v) regeneration of nickel(II) species.

**Scheme 41:**
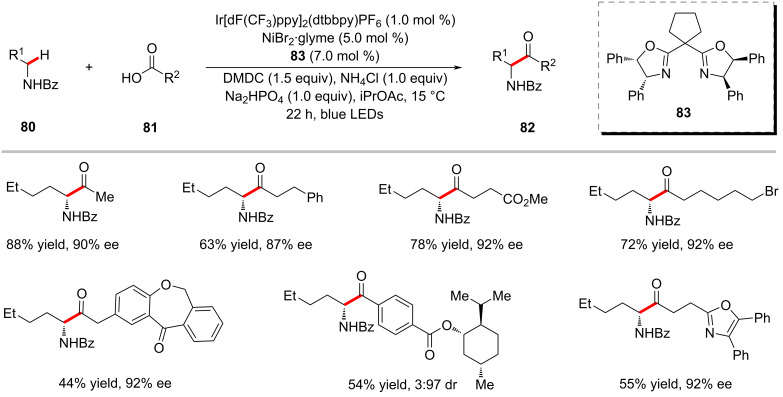
Photoredox nickel-catalyzed enantioselective acylation of α-amino C(sp^3^)–H bonds with carboxylic acids.

Recently, the group of Huo developed a nickel-catalyzed enantioselective acylation of α-amino C(sp^3^)–H bonds with carboxylic acids under visible light irradiation (Scheme 41) [124]. Here, dimethyl dicarbonate (DMDC) was the choice of activator to convert the carboxylic acid to a mixed anhydride in situ. In this protocol, the bisoxazoline-based chiral ligand **83** enabled the synthesis of α-amino ketones in high enantioselectivities under mild reaction conditions. Good yields were observed for carboxylic acid substrates **81** with different steric properties. Similarly, amine substrates **80** with diverse substitution patterns and functional groups were well tolerated to provide the desired products in optimal yields. The proposed mechanism involves the cleavage of the C(sp^3^)–H bond by a photo-generated bromine radical to give the carbon-centered alkyl radical, which subsequently engages in the nickel-catalyzed enantioselective acylation.

**Scheme 42:**
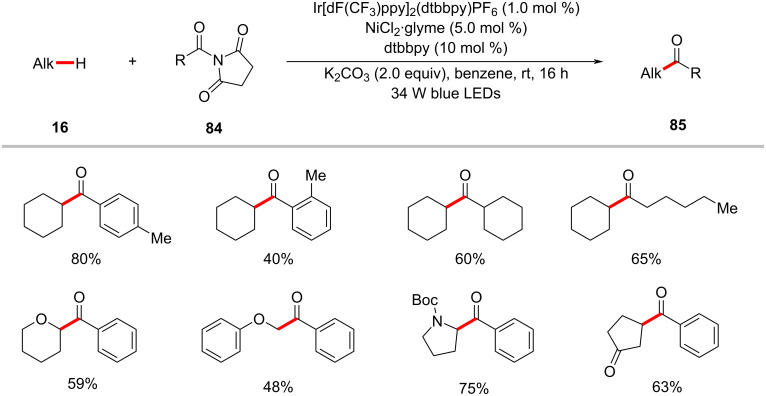
Nickel-catalyzed C(sp^3^)‒H acylation with *N*-acylsuccinimides.

**Figure 19:**
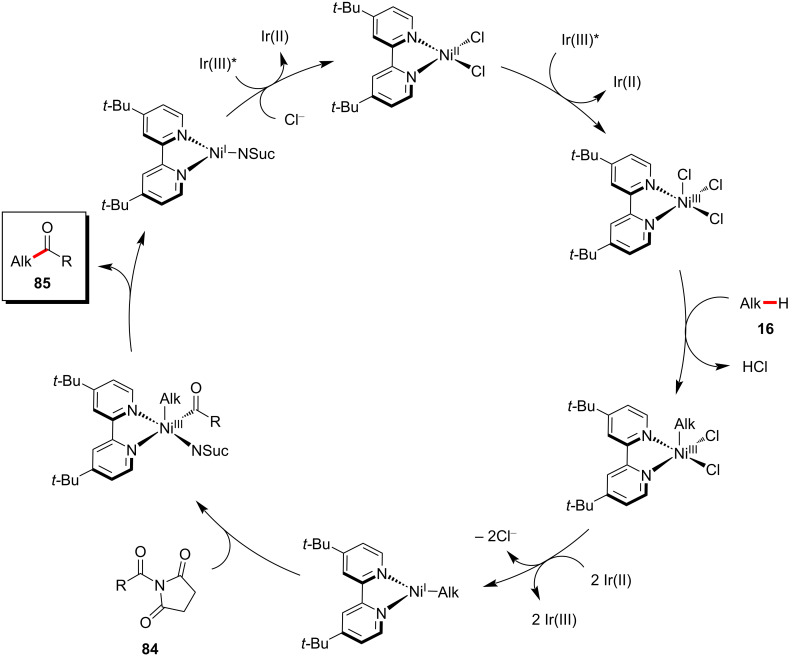
Proposed mechanism for the nickel-catalyzed C(sp^3^)–H acylation with *N*-acylsuccinimides.

Amides were also found to be competent acyl surrogates in the photoredox nickel-catalyzed direct C(sp^3^)–H acylation reactions as reported by Hong and co-workers (Scheme 42) [125]. Here, the two challenging bonds, the amide C‒N and alkane C(sp^3^)‒H were activated under mild photoredox reaction conditions. Among the various tested amides, *N*-acylsuccinimides **84** were found to be superior acyl surrogates to give the desired products **85** in high yields. Based on the detailed computational and experimental mechanistic studies, the authors proposed a catalytic cycle which involves the C–H cleavage prior to the oxidative addition of *N*-acylsuccinimide (Figure 19) [125].

**Scheme 43:**
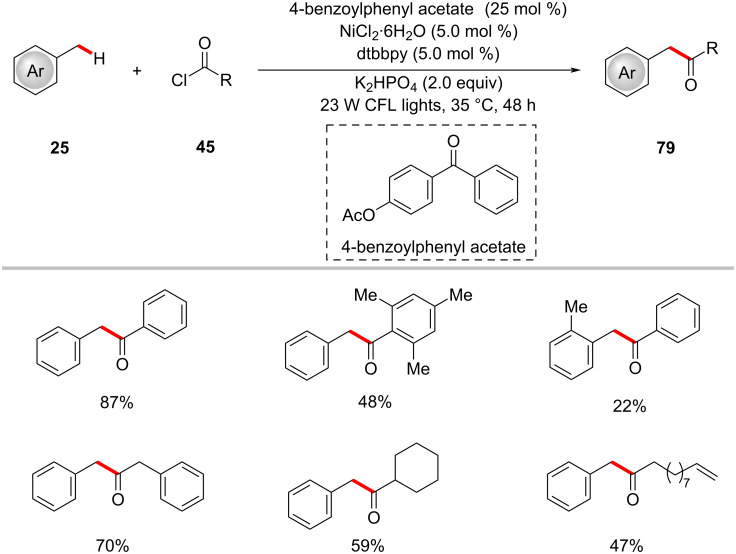
Nickel-catalyzed benzylic C–H functionalization with acid chlorides **45**.

The acylation of benzylic C‒H bonds with acid chlorides **45** by means of photoredox nickel catalysis was demonstrated by Rueping in 2020 (Scheme 43) [126]. Using substituted benzophenone 4-benzoylphenyl acetate as the photocatalyst, a variety of substituted methylbenzenes **25** were transformed into unsymmetrical ketones **79** under visible light irradiation. Both aromatic and aliphatic acid chlorides **45** were well tolerated under the catalytic conditions to offer the ketone products **79**. The authors also showed that acid anhydrides could also be used as viable acylating reagents under the optimized reaction conditions, however, with less efficacy than acid chlorides.

**Scheme 44:**
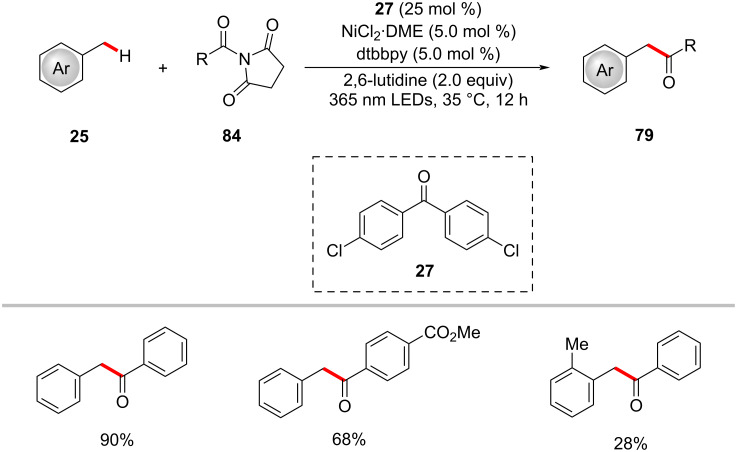
Photoredox nickel-catalyzed benzylic C–H acylation with *N*-acylsuccinimides **84**.

A related process involved the conversion of toluene into 1,2-arylethanone using 4,4’-dichlorobenzophenone (**27**) as the photocatalyst and NiCl_2_·DME as the nickel catalyst under UVA irradiation (Scheme 44) [127]. Here, *N*-acylsuccinimides **84** were used as the acyl source. Notably, *ortho*-substituted methylbenzenes gave lower yields due to steric effects.

**Scheme 45:**
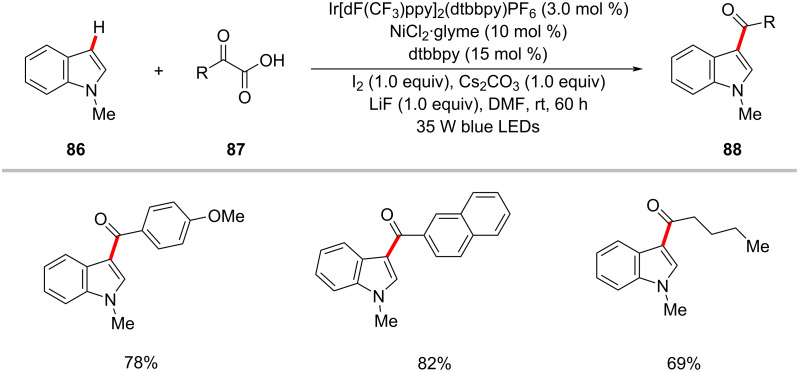
Photoredox nickel-catalyzed acylation of indoles **86** with α-oxoacids **87**.

The photoredox nickel-catalyzed C–H acylation was not limited to C(sp^3^)–H functionalization. Gu, Yuan and co-workers hence succeeded in preparing 3-acylindoles **88** from indole **86** and α-oxoacids **87** at room temperature by means of iridium photocatalysis and nickel catalysis under blue light irradiation (Scheme 45) [128]. Among the tested several commercially available photocatalysts, Ir[dF(CF_3_)ppy]_2_(dtbbpy)PF_6_ was found to provide the desired products in good yields.

### Aldehyde C–H functionalization

**Scheme 46:**
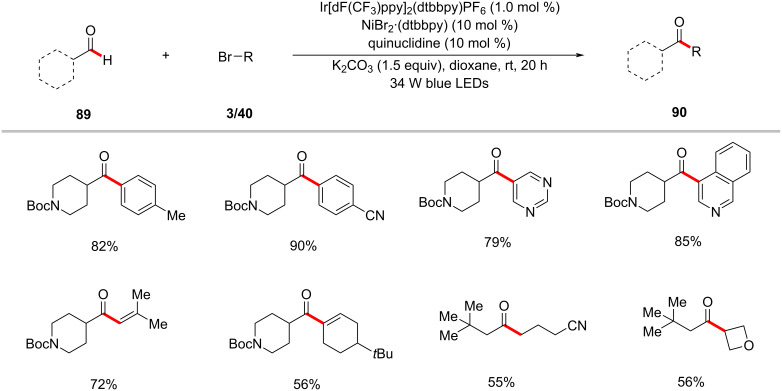
Nickel-catalyzed aldehyde C–H functionalization.

**Figure 20:**
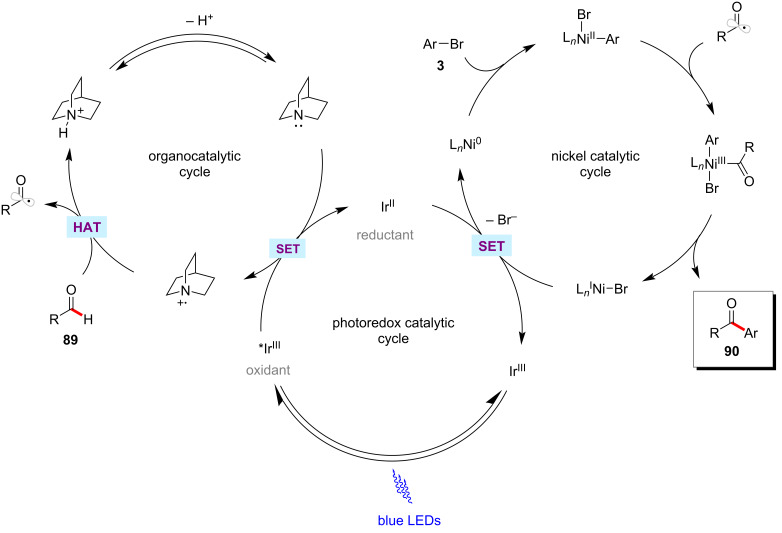
Proposed catalytic cycle for the photoredox nickel-catalyzed aldehyde C–H functionalization.

Inspired by their earlier contributions on HAT-metallaphotoredox-mediated C(sp^3^)–H functionalizations [53–54], the MacMillan group reported a photoredox nickel-catalyzed aldehyde C–H arylation, vinylation, or alkylation [129]. The ketone-forming reaction was conveniently realized by the reaction of aldehydes **89** with aryl, alkenyl, or alkyl bromides in the presence of Ir[dF(CF_3_)ppy]_2_(dtbbpy)PF_6_, NiBr_2_·dtbbpy, quinuclidine, and K_2_CO_3_ in dioxane under blue light irradiation at ambient reaction temperature (Scheme 46) [129]. Besides aryl bromides, alkenyl and alkyl bromides were found to be viable substrates and showcased the catalytic conditions versatility. Based on their experiments, the authors proposed a working mode for this protocol involving a triple catalysis mechanism (Figure 20) [129]. The synergistic merger of photoredox, nickel, and HAT catalytic cycles enabled the aldehyde C–H functionalization. Subsequently, a related transformation was also reported by Liu and co-workers [130]. Here, stoichiometric quantities of quinuclidine were used to get optimal results.

### Carboxylation

**Scheme 47:**
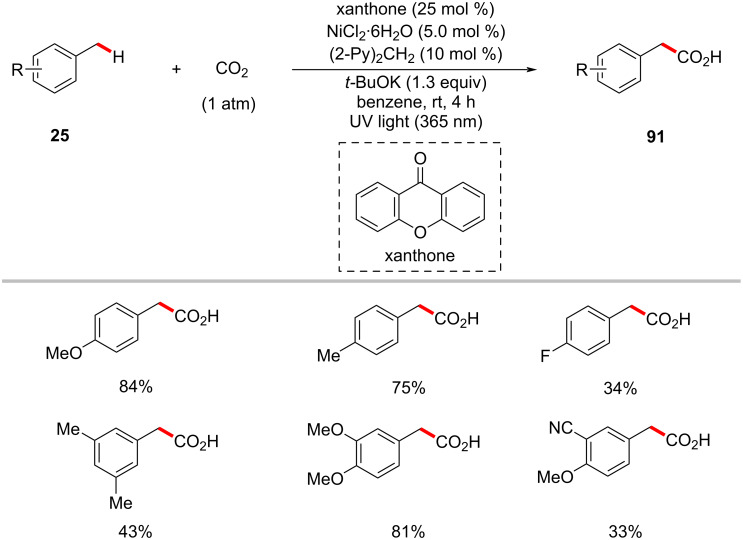
Photoredox carboxylation of methylbenzenes with CO_2_.

**Figure 21:**
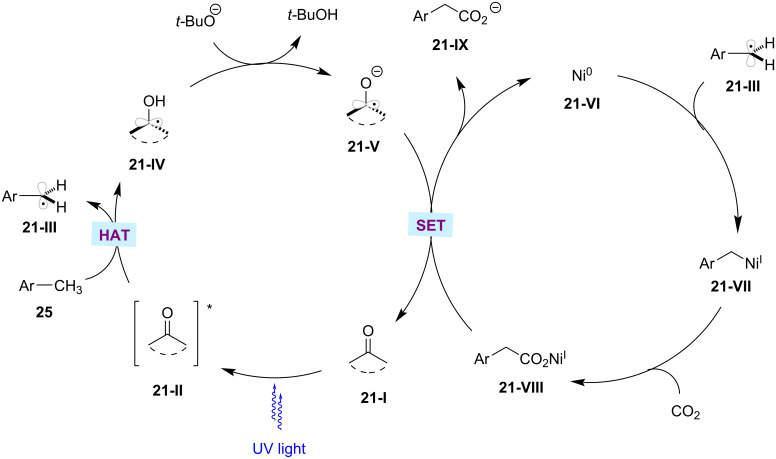
Proposed mechanism for the photoredox carboxylation of methylbenzenes with CO_2_.

Over the past few decades, significant attention has been devoted to exploit carbon dioxide (CO_2_) as the C1 resource [131–132]. In particular, the C–H functionalization with CO_2_ is considered an attractive organic synthesis strategy in terms of sustainable aspects [133–135]. In 2019, Murakami and co-workers reported on the photoinduced carboxylation of C(sp^3^)–H bonds with CO_2_ under 1 atm pressure [136]. Here, the authors discovered that the combination of xanthone as the photocatalyst and NiCl_2_·6H_2_O as the nickel catalyst can efficiently catalyze the transformation of methylarenes **25** into arylacetic acids **91** under UV light irradiation (Scheme 47). Furthermore, the authors also applied this methodology to functionalize unactivated alkanes such as cyclohexane, cyclopentanes, and *n*-pentane. The proposed catalytic cycle is initiated by the absorption of light by xanthone PC **21-I** to get excited (Figure 21) [136]. The excited ketone PC undergoes a HAT process with the benzylic C–H substrate to generate a pair of ketyl radical **21-IV** and benzylic radical **21-III**. At the same time, the in situ-generated nickel(0) complex **21-VI** combines with the benzylic radical **21-III**, followed by CO_2_ insertion resulting in the nickel(I) carboxylate complex **21-VIII**. The ketyl radical is deprotonated by the base, and then undergoes a SET with nickel(I) carboxylate complex **21-VIII** to regenerate the nickel(0) species **21-VI** and the carboxylate product **21-IX**.

### Olefin difunctionalization

**Scheme 48:**
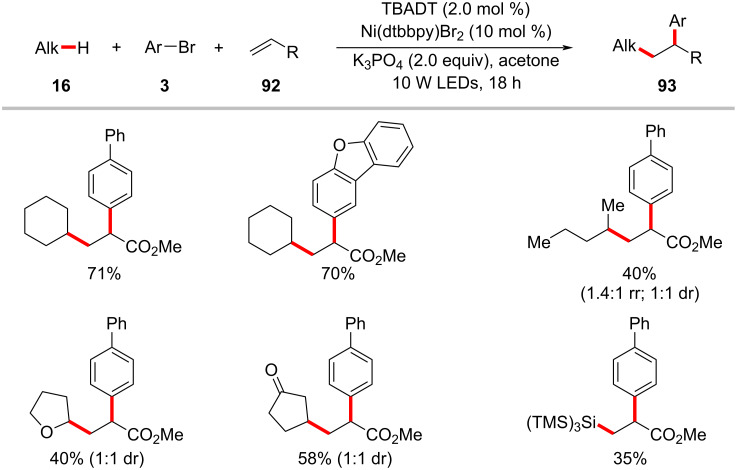
Decatungstate photo-HAT and nickel catalysis enabled alkene difunctionalization.

**Figure 22:**
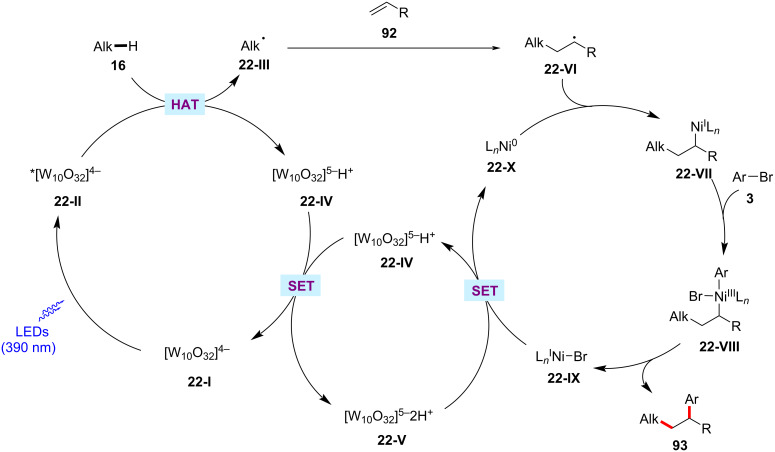
Proposed catalytic cycle for the decatungstate photo-HAT and nickel catalysis enabled alkene difunctionalization.

Nickel-catalyzed alkene 1,2-difunctionalization is considered as useful method for preparing complex molecules in a single-step reaction [137–139]. In this aspect, the groups by Kong [140] and Molander [141] independently demonstrated photoredox/nickel-catalyzed approaches to olefin difunctionalizations involving C(sp^3^)–H activation. Thus, Kong devised a synthetic method combining nickel catalysis with tetrabutylammonium decatungstate (TBADT) as photocatalyst for the three component reaction of alkanes **16**, alkenes **92**, and aryl bromides **3** (Scheme 48) [140]. Here, TBADT enables the generation of alkyl radicals from various alkane substrates via a HAT process under near-ultraviolet light irradiation. Both cyclic and linear alkanes were found to be suitable under the reaction conditions. Linear alkanes were preferentially functionalized at the 2-position due to the less steric hindrance. In addition to alkanes, a variety of ethers and amines were also compatible and selectively functionalized at the α-heteroatom positions in moderate to good yields and excellent regioselectivity. Interestingly, ketones and silanes were also found to be compatible to give the desired three-component coupling products. Similarly, the scope of aryl bromides **3** and alkenes **92** were found to be broad. A possible catalytic cycle was proposed to account for the mechanism of the reaction (Figure 22) [140]. Photoexcited decatungstate **22-II** undergoes a HAT process with the C(sp^3^)–H substrate to form a carbon-centered radical species **22-III** and reduced decatungstate **22-IV**. The thus formed alkyl radical **22-III** adds to the alkene **92** affording the radical adduct **22-VI**, which is intercepted by the nickel(0) species **22-X** to generate alkyl-nickel(I) intermediate **22-VII**. Oxidative addition of the aryl bromide **3** to intermediate **22-VII** results in (alkyl)(aryl)nickel(III) intermediate **22-VIII**, which subsequently undergoes reductive elimination to deliver the desired cross-coupled product **93** and the nickel(I) species **22-IX**. A SET process between **22-IX** and **22-V** regenerates the reduced decatungstate **22-IV** and the active nickel(0) catalyst **22-X**.

**Scheme 49:**
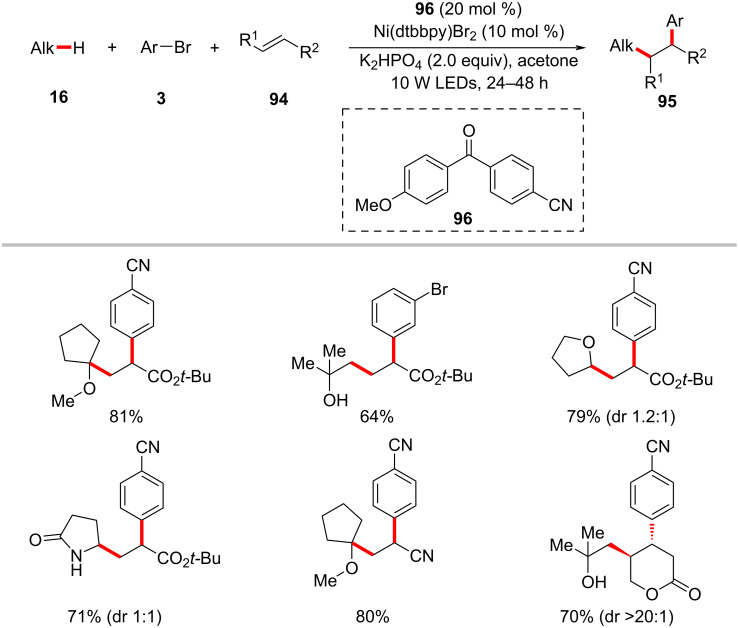
Diaryl ketone HAT catalysis and nickel catalysis enabled dicarbofunctionalization of alkenes.

**Figure 23:**
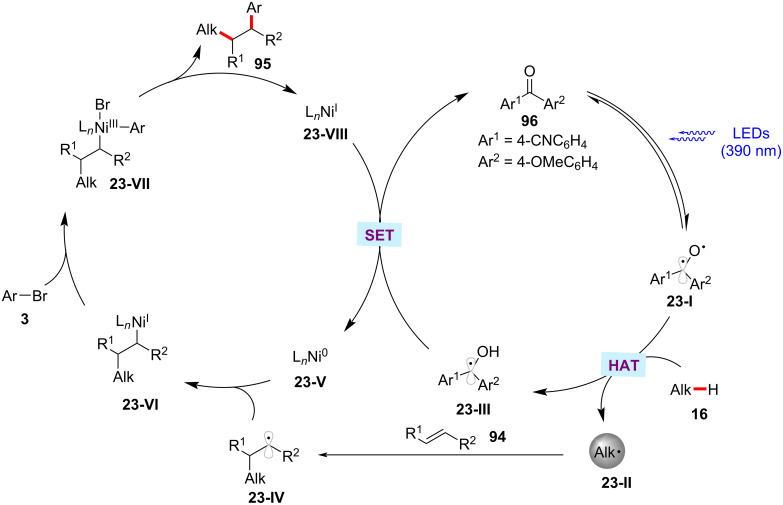
Proposed catalytic mechanism for the diaryl ketone HAT catalysis and nickel catalysis enabled dicarbofunctionalization of alkenes.

In a recent report, Gutierrez and Molander realized the three-component dicarbofunctionalization of alkenes by means of the combination of phororedox HAT catalysis and nickel catalysis [141]. Here, a substituted diaryl ketone, 4-(4-methoxybenzoyl)benzonitrile (**96**) serves as the HAT photocatalyst to activate the C(sp^3^)–H bonds for olefin functionalization. It was identified that the use of nonpolar, aprotic solvents, such as benzene and α,α,α-trifluorotoluene (TFT) is critical for the formation of the desired products **95**. The scope of the transformation was demonstrated with a variety of activated alkenes **94**, alkyl C‒H substrates **16**, and aryl bromides **3**. In general, the products were obtained in moderate to good yields and good regioselectivities (Scheme 49) [141]. The detailed experimental and computational studies highlight the involvement of hydrogen bonding assistance during the radical addition to olefine. The proposed reaction mechanism has two synergistic catalytic cycles, namely a photocatalytic cycle and a nickel catalytic cycle (Figure 23). The photoexcitation of the ketone PC **96** results in the triplet-state diradical **23-I**. A HAT process between **23-I** and the alkane substrate generates the desired carbon-centered radical **23-II** with concomitant formation of ketyl radical species **23-III**. The thus formed alkyl radical **23-II** undergoes Giese addition to alkene **94** resulting in the radical adduct **23-IV**. The radical adduct **23-IV** is captured by nickel(0) species **23-V** followed by oxidative addition to aryl bromide **3** to give the nickel(III)(alkyl)(aryl) intermediate **23-VII**. Facile C–C-bond forming reductive elimination of **23-VII** delivers the desired product **95** and nickel(I) species **23-VIII**. A SET between **23-VIII** and **23-III** regenerates the active nickel(0) species **23-V** and the ketone PC **96**. Alternatively, the nickel(III) intermediate **23-VII** could also be formed via an oxidative addition of the nickel(0) species **23-V** to aryl bromide **3** followed by the reaction with alkyl radical **23-IV**.

## Conclusion

**Scheme 50:**
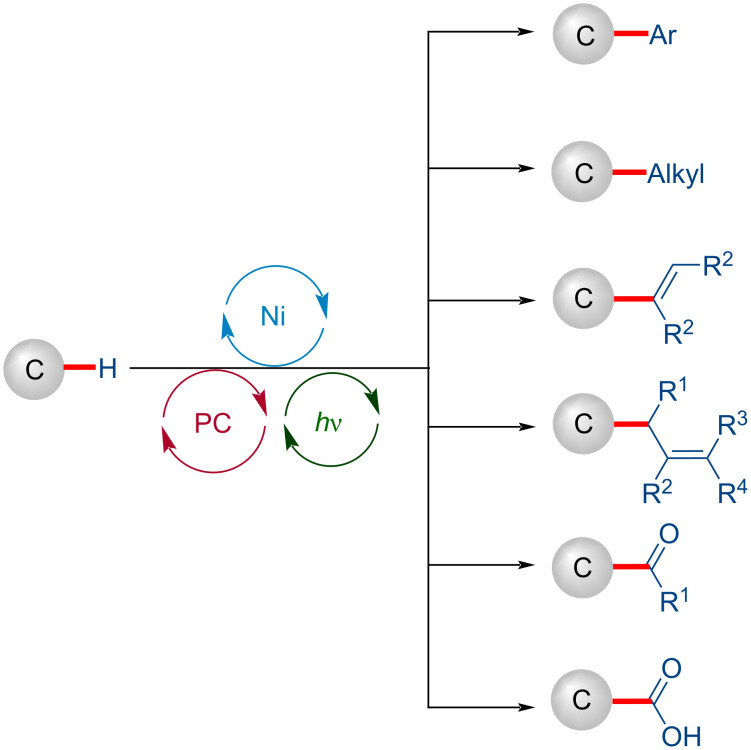
Overview of photoredox nickel-catalyzed C–H functionalizations.

During the last decade, metallaphotoredox catalysis has emerged as an increasingly viable tool in organic synthesis for C–H functionalization. Although significant advances have been achieved with precious palladium catalysts, recently, considerable attention has been devoted to using earth-abundant, less toxic, and cost-effective nickel catalysts. It is clear from the wealth of the different transformations discussed in this review, the merger of photoredox catalysis and nickel catalysis offers a range of new tools for organic synthesis (Scheme 50). The impressive array of transformations involving C(sp^3^)–H functionalizations, including arylation, alkylation, alkenylation, allylation, acylation, and carboxylation, highlights their potential utility in organic synthesis. Further, the mild nature of the reaction conditions enables a broad substrate scope, functional group tolerance, and opportunities for late-stage diversification of complex molecules. Despite the signiﬁcant advances, the photoredox-mediated nickel-catalyzed C‒H functionalization is still in its infancy. Thus far, expensive iridium-based complexes are the most common photocatalysts and are essential to achieve satisfactory outcomes; less expensive organic photocatalysts in nickel-catalyzed transformations are less explored. Further, the major challenges of C‒H functionalization, including site speciﬁcity and functionalization of stronger C–H bonds, remain unexplored. Furthermore, examples of enantioselective C–H functionalizations are scarce and present new opportunities for further exploration. In consideration of the sustainable nature of C–H activation by photoredox nickel catalysis, further exciting developments are expected in this rapidly evolving research area.

## References

[1] Boström J, Brown D G, Young R J, Keserü G M (2018). Nat Rev Drug Discovery.

[2] Dreher S D (2019). React Chem Eng.

[3] Blakemore D C, Castro L, Churcher I, Rees D C, Thomas A W, Wilson D M, Wood A (2018). Nat Chem.

[4] Hayler J D, Leahy D K, Simmons E M (2019). Organometallics.

[5] Johansson Seechurn C C C, Kitching M O, Colacot T J, Snieckus V (2012). Angew Chem, Int Ed.

[6] Heck R F (2006). Synlett.

[7] Heck R F (1982). Org React.

[8] Mizoroki T, Mori K, Ozaki A (1971). Bull Chem Soc Jpn.

[9] Suzuki A (2011). Angew Chem, Int Ed.

[10] Miyaura N, Suzuki A (1995). Chem Rev.

[11] Suzuki A (1999). J Organomet Chem.

[12] Ruiz-Castillo P, Buchwald S L (2016). Chem Rev.

[13] Hartwig J F (2008). Acc Chem Res.

[14] Negishi E-i (2011). Angew Chem, Int Ed.

[15] Negishi E (1982). Acc Chem Res.

[16] Stille J K (1986). Angew Chem, Int Ed Engl.

[17] Sonogashira K, Tohda Y, Hagihara N (1975). Tetrahedron Lett.

[18] de Meijere A, Diederich F (2004). Metal-Catalyzed Cross-Coupling Reactions.

[19] Diederich F, Stang P J (1998). Metal-Catalyzed Cross-Coupling Reactions.

[20] Ackermann L (2009). Modern Arylation Methods.

[21] Brown D G, Boström J (2016). J Med Chem.

[22] Buskes M J, Blanco M-J (2020). Molecules.

[23] Diccianni J B, Diao T (2019). Trends Chem.

[24] Shi R, Zhang Z, Hu X (2019). Acc Chem Res.

[25] Iwasaki T, Kambe N (2016). Top Curr Chem.

[26] Gandeepan P, Müller T, Zell D, Cera G, Warratz S, Ackermann L (2019). Chem Rev.

[27] Yamaguchi J, Muto K, Itami K (2016). Top Curr Chem.

[28] Castro L C M, Chatani N (2015). Chem Lett.

[29] Zhang S-K, Samanta R C, Del Vecchio A, Ackermann L (2020). Chem – Eur J.

[30] Tasker S Z, Standley E A, Jamison T F (2014). Nature.

[31] Fagnoni M, Dondi D, Ravelli D, Albini A (2007). Chem Rev.

[32] Narayanam J M R, Stephenson C R J (2011). Chem Soc Rev.

[33] Xuan J, Xiao W-J (2012). Angew Chem, Int Ed.

[34] Prier C K, Rankic D A, MacMillan D W C (2013). Chem Rev.

[35] Xi Y, Yi H, Lei A (2013). Org Biomol Chem.

[36] Shaw M H, Twilton J, MacMillan D W C (2016). J Org Chem.

[37] Romero N A, Nicewicz D A (2016). Chem Rev.

[38] Schultz D M, Yoon T P (2014). Science.

[39] Strieth-Kalthoff F, James M J, Teders M, Pitzer L, Glorius F (2018). Chem Soc Rev.

[40] Guillemard L, Wencel-Delord J (2020). Beilstein J Org Chem.

[41] Milligan J A, Phelan J P, Badir S O, Molander G A (2019). Angew Chem, Int Ed.

[42] Cavalcanti L N, Molander G A (2016). Top Curr Chem.

[43] Wenger O S (2021). Chem – Eur J.

[44] Kariofillis S K, Doyle A G (2021). Acc Chem Res.

[45] Capaldo L, Quadri L L, Ravelli D (2020). Green Chem.

[46] Dwivedi V, Kalsi D, Sundararaju B (2019). ChemCatChem.

[47] Twilton J, Le C, Zhang P, Shaw M H, Evans R W, MacMillan D W C (2017). Nat Rev Chem.

[48] Caplin M J, Foley D J (2021). Chem Sci.

[49] Das J, Guin S, Maiti D (2020). Chem Sci.

[50] He C, Whitehurst W G, Gaunt M J (2019). Chem.

[51] Saint-Denis T G, Zhu R-Y, Chen G, Wu Q-F, Yu J-Q (2018). Science.

[52] Baudoin O (2011). Chem Soc Rev.

[53] Zuo Z, Ahneman D T, Chu L, Terrett J A, Doyle A G, MacMillan D W C (2014). Science.

[54] Shaw M H, Shurtleff V W, Terrett J A, Cuthbertson J D, MacMillan D W C (2016). Science.

[55] Ahneman D T, Doyle A G (2016). Chem Sci.

[56] Shields B J, Doyle A G (2016). J Am Chem Soc.

[57] Heitz D R, Tellis J C, Molander G A (2016). J Am Chem Soc.

[58] Gui Y-Y, Liao L-L, Sun L, Zhang Z, Ye J-H, Shen G, Lu Z-P, Zhou W-J, Yu D-G (2017). Chem Commun.

[59] Gui Y-Y, Chen X-W, Zhou W-J, Yu D-G (2017). Synlett.

[60] Gui Y-Y, Wang Z-X, Zhou W-J, Liao L-L, Song L, Yin Z-B, Li J, Yu D-G (2018). Asian J Org Chem.

[61] Nielsen M K, Shields B J, Liu J, Williams M J, Zacuto M J, Doyle A G (2017). Angew Chem, Int Ed.

[62] Perry I B, Brewer T F, Sarver P J, Schultz D M, DiRocco D A, MacMillan D W C (2018). Nature.

[63] Twilton J, Christensen M, DiRocco D A, Ruck R T, Davies I W, MacMillan D W C (2018). Angew Chem, Int Ed.

[64] Huang L, Rueping M (2018). Angew Chem, Int Ed.

[65] Zhao J, Wu W, Sun J, Guo S (2013). Chem Soc Rev.

[66] Shen Y, Gu Y, Martin R (2018). J Am Chem Soc.

[67] Dewanji A, Krach P E, Rueping M (2019). Angew Chem, Int Ed.

[68] Si X, Zhang L, Hashmi A S K (2019). Org Lett.

[69] Loup J, Dhawa U, Pesciaioli F, Wencel‐Delord J, Ackermann L (2019). Angew Chem, Int Ed.

[70] Woźniak Ł, Cramer N (2019). Trends Chem.

[71] Cheng X, Lu H, Lu Z (2019). Nat Commun.

[72] Rand A W, Yin H, Xu L, Giacoboni J, Martin-Montero R, Romano C, Montgomery J, Martin R (2020). ACS Catal.

[73] Li H, Guo L, Feng X, Huo L, Zhu S, Chu L (2020). Chem Sci.

[74] Xiao J, Liu X, Pan L, Shi C, Zhang X, Zou J-J (2020). ACS Catal.

[75] Mazzanti S, Savateev A (2020). ChemPlusChem.

[76] Gisbertz S, Pieber B (2020). ChemPhotoChem.

[77] Das S, Murugesan K, Villegas Rodríguez G J, Kaur J, Barham J P, Savateev A, Antonietti M, König B (2021). ACS Catal.

[78] Peng L, Li Z, Yin G (2018). Org Lett.

[79] Evano G, Theunissen C (2019). Angew Chem, Int Ed.

[80] Ankade S B, Shabade A B, Soni V, Punji B (2021). ACS Catal.

[81] Ackermann L (2010). Chem Commun.

[82] Chen Z, Rong M-Y, Nie J, Zhu X-F, Shi B-F, Ma J-A (2019). Chem Soc Rev.

[83] Kwiatkowski M R, Alexanian E J (2019). Acc Chem Res.

[84] Choi J, Fu G C (2017). Science.

[85] Le C, Liang Y, Evans R W, Li X, MacMillan D W C (2017). Nature.

[86] Zhang L, Si X, Yang Y, Zimmer M, Witzel S, Sekine K, Rudolph M, Hashmi A S K (2019). Angew Chem, Int Ed.

[87] Santos M S, Corrêa A G, Paixão M W, König B (2020). Adv Synth Catal.

[88] Schönherr H, Cernak T (2013). Angew Chem, Int Ed.

[89] Yan G, Borah A J, Wang L, Yang M (2015). Adv Synth Catal.

[90] Friis S D, Johansson M J, Ackermann L (2020). Nat Chem.

[91] Aynetdinova D, Callens M C, Hicks H B, Poh C Y X, Shennan B D A, Boyd A M, Lim Z H, Leitch J A, Dixon D J (2021). Chem Soc Rev.

[92] Kariofillis S K, Shields B J, Tekle-Smith M A, Zacuto M J, Doyle A G (2020). J Am Chem Soc.

[93] Vasilopoulos A, Krska S W, Stahl S S (2021). Science.

[94] Thullen S M, Treacy S M, Rovis T (2019). J Am Chem Soc.

[95] Ali W, Prakash G, Maiti D (2021). Chem Sci.

[96] Vivek Kumar S, Banerjee S, Punniyamurthy T (2020). Org Chem Front.

[97] Ma W, Gandeepan P, Li J, Ackermann L (2017). Org Chem Front.

[98] Manikandan R, Jeganmohan M (2017). Chem Commun.

[99] Kozhushkov S I, Ackermann L (2013). Chem Sci.

[100] Le Bras J, Muzart J (2011). Chem Rev.

[101] Gandeepan P, Ackermann L, Colobert F, Wencel‐Delord J (2019). Diastereoselective formation of alkenes through C(sp2)–H bond activation. C–H Activation for Asymmetric Synthesis.

[102] Mishra A A, Subhedar D, Bhanage B M (2019). Chem Rec.

[103] Lim H N, Xing D, Dong G (2019). Synlett.

[104] Gonnard L, Guérinot A, Cossy J (2019). Tetrahedron.

[105] Antermite D, Bull J A (2019). Synthesis.

[106] Chu J C K, Rovis T (2018). Angew Chem, Int Ed.

[107] Deng H-P, Fan X-Z, Chen Z-H, Xu Q-H, Wu J (2017). J Am Chem Soc.

[108] Go S Y, Lee G S, Hong S H (2018). Org Lett.

[109] Mishra N K, Sharma S, Park J, Han S, Kim I S (2017). ACS Catal.

[110] Wu K, Wang L, Colón‐Rodríguez S, Flechsig G-U, Wang T (2019). Angew Chem, Int Ed.

[111] Shu W, Genoux A, Li Z, Nevado C (2017). Angew Chem, Int Ed.

[112] Xu B, Tambar U K (2019). ACS Catal.

[113] Yue W-J, Day C S, Martin R (2021). J Am Chem Soc.

[114] Rajamalli P, Senthilkumar N, Gandeepan P, Huang P-Y, Huang M-J, Ren-Wu C-Z, Yang C-Y, Chiu M-J, Chu L-K, Lin H-W (2016). J Am Chem Soc.

[115] Carroll F I, Blough B E, Abraham P, Mills A C, Holleman J A, Wolckenhauer S A, Decker A M, Landavazo A, McElroy K T, Navarro H A (2009). J Med Chem.

[116] Meltzer P C, Butler D, Deschamps J R, Madras B K (2006). J Med Chem.

[117] Foley K F, Cozzi N V (2003). Drug Dev Res.

[118] Penteado F, Lopes E F, Alves D, Perin G, Jacob R G, Lenardão E J (2019). Chem Rev.

[119] Wu X-F (2015). Chem – Eur J.

[120] Joe C L, Doyle A G (2016). Angew Chem, Int Ed.

[121] Sun Z, Kumagai N, Shibasaki M (2017). Org Lett.

[122] Ackerman L K G, Martinez Alvarado J I, Doyle A G (2018). J Am Chem Soc.

[123] Kawasaki T, Ishida N, Murakami M (2020). J Am Chem Soc.

[124] Shu X, Huan L, Huang Q, Huo H (2020). J Am Chem Soc.

[125] Lee G S, Won J, Choi S, Baik M-H, Hong S H (2020). Angew Chem, Int Ed.

[126] Krach P E, Dewanji A, Yuan T, Rueping M (2020). Chem Commun.

[127] Ren C-C, Wang T-Q, Zhang Y, Peng D, Liu X-Q, Wu Q-A, Liu X-F, Luo S-P (2021). ChemistrySelect.

[128] Gu L, Jin C, Liu J, Zhang H, Yuan M, Li G (2016). Green Chem.

[129] Zhang X, MacMillan D W C (2017). J Am Chem Soc.

[130] Vu M D, Das M, Liu X-W (2017). Chem – Eur J.

[131] Artz J, Müller T E, Thenert K, Kleinekorte J, Meys R, Sternberg A, Bardow A, Leitner W (2018). Chem Rev.

[132] Sakakura T, Choi J-C, Yasuda H (2007). Chem Rev.

[133] Luo J, Larrosa I (2017). ChemSusChem.

[134] Kleij A, Rintjema J (2016). Synthesis.

[135] Yeung C S, Dong V M (2014). Top Catal.

[136] Ishida N, Masuda Y, Imamura Y, Yamazaki K, Murakami M (2019). J Am Chem Soc.

[137] Bag D, Mahajan S, Sawant S D (2020). Adv Synth Catal.

[138] Derosa J, Apolinar O, Kang T, Tran V T, Engle K M (2020). Chem Sci.

[139] Qi X, Diao T (2020). ACS Catal.

[140] Xu S, Chen H, Zhou Z, Kong W (2021). Angew Chem, Int Ed.

[141] Campbell M W, Yuan M, Polites V C, Gutierrez O, Molander G A (2021). J Am Chem Soc.

